# The regulatory mechanism of NLRP3 inflammasome in the “immune paralytic-overactivation” imbalance in sepsis: the latest progress from molecular signaling to clinical translation

**DOI:** 10.3389/fimmu.2026.1751283

**Published:** 2026-04-30

**Authors:** Gaosheng Zhou, Yifei Chen, Dingdeng Wang, Yixin Cheng, Zhaohui Zhang

**Affiliations:** 1 Critical Care Medicine Laboratory, Yichang Sepsis Clinical Research Center, Yichang, Hubei, China; 2 Laboratory of Critical Care Medicine, Hubei Provincial Clinical Research Center for Critical Care Medicine (Sepsis Research Collaborative Unit), Yichang, Hubei, China; 3 Medical Administration Department, Yichang Central People’s Hospital, Yichang, Hubei, China; 4 The First College of Clinical Medical Science, China Three Gorges University, Yichang, Hubei, China; 5 Department of Critical Care Medicine, Yichang Central People’s Hospital, Yichang, Hubei, China

**Keywords:** clinical translation, immune hyperactivation, immunoparalysis, molecular mechanism, NLRP3 inflammasome, sepsis

## Abstract

Sepsis remains a major challenge in critical care medicine worldwide, characterized by a dynamic imbalance of the immune system between two extremes: “immunoparalysis” and “hyperactivation.” This dysregulation severely affects patient survival. The NLRP3 inflammasome, a central molecular platform in innate immunity, has recently been shown to play a critical dual role in the pathogenesis of sepsis. This review systematically outlines the structural features and activation mechanisms of the NLRP3 inflammasome, elaborates on its pro-inflammatory and immunosuppressive effects in sepsis-induced immune dysregulation, and summarizes the associated signaling pathways and regulatory networks. By integrating recent advances in basic and clinical research, we provide an in-depth analysis of the molecular regulatory mechanisms of the NLRP3 inflammasome in sepsis and evaluate its potential as a therapeutic target. Furthermore, this review discusses the opportunities and challenges in translating NLRP3-targeted strategies into clinical practice, emphasizing the potential of precise NLRP3 modulation to restore immune homeostasis in sepsis. Our findings may provide a theoretical foundation and future research directions for developing novel therapeutic approaches.

## 1 Introduction

Sepsis, a systemic inflammatory response syndrome triggered by infection, remains a major global health concern with, affecting an estimated 48.9 million people annually and contributing to 11 million deaths worldwide, which represent approximately 19.7% of all global deaths (1–5). Although advances in critical care and antimicrobial therapy have reduced case fatality in some regions, the overall clinical outcome remains poor. Diagnosis and treatment continue to pose significant challenges, particularly in resource-limited settings (6, 7). The highly heterogeneous clinical presentation of sepsis often involves multiple organ dysfunction, contributing to unfavorable prognoses and substantial healthcare costs (8, 9).

**Figure 1:**
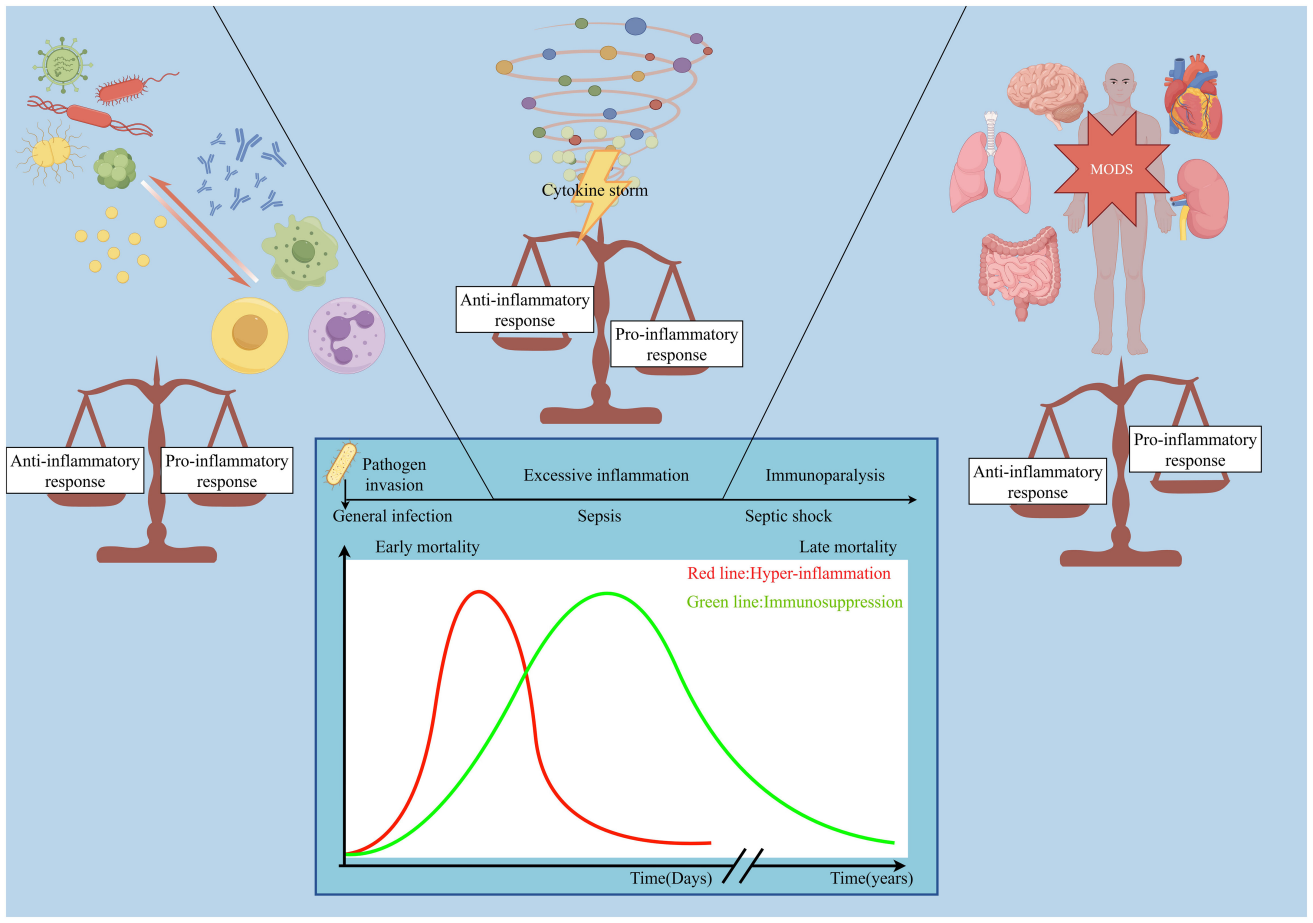
The dynamic immunological trajectory of sepsis. Following pathogen invasion, an initial hyperinflammatory phase, characterized by a cytokine storm (red line), can precipitate septic shock and early mortality. This phase is often followed by a protracted state of immunosuppression (green line), marked by immunoparalysis, which increases susceptibility to secondary infections, multiple organ dysfunction syndrome (MODS), and late mortality. The image was generated using Figdraw.

Immune dysregulation is considered a central mechanism driving the progression and worsening outcomes of sepsis. Patients often experience a dynamic shift between two extreme immune states (**Figure 1**): early hyperinflammation, characterized by a cytokine storm and systemic inflammatory response syndrome (SIRS), leading to tissue injury and organ failure; and a subsequent immunosuppressive phase, marked by impaired immune cell function, increased susceptibility to secondary infections, and failure to clear pathogens (10, 11). This immune imbalance not only shapes the clinical course and outcome of sepsis but also represents a major obstacle to effective treatment (12).

In recent years, the NOD-like receptor protein 3 (NLRP3) inflammasome, a key regulator of inflammatory responses, has gained increasing attention for its role in sepsis-associated immune dysregulation. The NLRP3 inflammasome is a multiprotein complex that senses various pathogen-associated molecular patterns (PAMPs) and damage-associated molecular patterns (DAMPs). Upon activation, it cleaves caspase-1, promoting the cleavage and secretion of proinflammatory cytokines such as IL-1β and IL-18, and inducing pyroptosis (13). Aberrant NLRP3 inflammasome activation has been implicated not only in the early inflammatory storm but also in late-stage immune dysfunction, reflecting its dual role in regulating the balance between immunoparalysis and hyperactivation (10, 11). Recent studies have further elucidated the signaling pathways and regulatory mechanisms of the NLRP3 inflammasome activation, its contribution to sepsis-induced organ injury, and its potential as a drug target (14). Several NLRP3 inhibitors have shown promising anti-inflammatory and organ-protective effects in animal models and early-phase clinical trials, highlighting their potential for precision therapy in sepsis (14, 15).

This review systematically outlines the regulatory mechanisms of the NLRP3 inflammasome in the immune paralysis–hyperactivation imbalance during sepsis, with a focus on its molecular signaling, immunomodulatory roles, and recent advances in clinical translation. We also discuss future prospects for targeting the NLRP3 inflammasome in the clinical management of sepsis.

## 2 Structure and activation mechanism of the NLRP3 inflammasome

### 2.1 Composition of the NLRP3 inflammasome

The NLRP3 inflammasome is a critical multiprotein complex in the innate immune system, primarily composed of NLRP3, apoptosis-associated speck-like protein containing a CARD (ASC), and caspase-1. NLRP3, a member of the NLR family, serves as the core sensor and consists of three domains: an N-terminal pyrin domain (PYD), a central NACHT domain, and C-terminal LRRs. The PYD mediates homotypic interaction with the PYD of ASC; the NACHT domain facilitates NLRP3 oligomerization and ATP binding/hydrolysis; and the LRR domain is involved in autoinhibition and ligand recognition, thereby modulating the activation status of NLRP3 (16–18).

ASC functions as an adaptor protein, bridging NLRP3 and caspase-1. Oligomerization of ASC leads to the formation of a filamentous “speck”, which is essential for inflammasome assembly and signal amplification (19). Studies have shown that ASC released into the extracellular space following pyroptosis can be phagocytosed by adjacent macrophages (20), where it reinitiates inflammasome assembly and caspase-1 activation within recipient cells by inducing phagosomal membrane damage and exerting an intracellular “seeding” effect (21). These findings suggest that ASC specks may serve as potential biomarkers and therapeutic targets in inflammatory diseases (22, 23).

Caspase-1, the effector component, is recruited as pro-caspase-1 via ASC and undergoes autocleavage into its active form. Activated caspase-1 processes pro-IL-1β and pro-IL-18 into their mature forms and cleaves gasdermin D to induce pyroptosis, a proinflammatory form of cell death (24, 25).The assembly of the NLRP3 inflammasome is spatiotemporally regulated. Upon sensing danger signals, the NLRP3 inflammasome undergoes conformational changes that expose its PYD, enabling ASC recruitment. Subsequent ASC oligomerization recruits pro-caspase-1, leading to the formation of a functional inflammasome complex (26).

### 2.2 Activation signals and molecular mechanisms

The activation of the NLRP3 inflammasome is a tightly regulated, multi-step process involving two main phases: priming and activation. In the priming phase, inflammatory stimuli such as PAMPs and DAMPs activate transcription factors including NF-κB, upregulating the expression of NLRP3 and its downstream components, thereby priming the cell for inflammasome assembly (27, 28). Studies indicate that NF-κB not only controls the transcription of NLRP3 mRNA and protein but also contributes to a feedforward inflammatory loop by regulating the expression of related cytokines (29). In various sepsis models, NF-κB inhibitors downregulate NLRP3 expression, attenuating inflammation and organ injury (30, 31).

The subsequent activation phase is triggered by diverse intracellular and extracellular signals, including ion flux (e.g., K^+^ efflux, Ca²^+^ influx), mitochondrial dysfunction, reactive oxygen species (ROS) production, lysosomal rupture, and mitochondrial DNA release (32–34). These stimuli promote the assembly of the NLRP3 inflammasome complex, which serves as a platform for pro-caspase-1 recruitment. Once recruited, pro-caspase-1 undergoes autoproteolytic cleavage to generate active caspase-1, which then processes pro-IL-1β and pro-IL-18 into their mature forms (35).

Mitochondrial impairment, for example, leads to the release of oxidized mitochondrial DNA into the cytosol, where it binds directly to the NLRP3 inflammasome and promotes its activation (33, 34). Damaged mitochondria produce excessive ROS, which not only cause direct cellular damage but also act as signaling molecules promoting the NLRP3 inflammasome activation (36–38).Ion channel perturbations also represent a major trigger for inflammasome assembly (39). K^+^ efflux has been identified as a common event triggered by multiple NLRP3 activators and is considered necessary for inflammasome assembly (40–42). Similarly, TRPM2 and TRPM7 contribute to inflammasome-mediated pyroptosis and tissue injury by modulating Ca²^+^ signaling (43, 44). Acid-sensing ion channels (ASICs), activated under extracellular acidification, promote Ca²^+^ influx and upregulate NLRP3 expression via NF-κB and other pathways, thereby amplifying inflammation (45, 46). Other changes in the intracellular environment—such as acidification (47), osmotic variation, and fluctuations in Na^+^, Cl^-^, or Mg²^+^—also modulate NLRP3 inflammasome activation (48–50).Furthermore, specific domains of NLRP3 inflammasome exert distinct regulatory functions in response to different activators, enabling the integration of varied inflammatory signals (51, 52).

The activation and stability of NLRP3 inflammasome and associated components are also finely tuned by post-translational modifications—such as ubiquitination, SUMOylation, and phosphorylation—which can either positively or negatively regulate inflammasome activation by influencing the localization, stability, and functional state of NLRP3 inflammasome (35, 53, 54).

In addition to the classical pathway described above, the non-canonical inflammasome pathway plays a critical role in disease progression during sepsis caused by Gram-negative bacteria (55, 56). This pathway is directly mediated by human caspase-4/5/11. When Gram-negative bacteria escape into the cytosol or deliver LPS into the intracellular compartment via outer membrane vesicles, the non-canonical pathway promotes NLRP3 inflammasome activation through two distinct mechanisms: it induces GSDMD pore formation leading to K^+^ efflux, which indirectly activates NLRP3; additionally, caspase-4/5/11 can directly facilitate NLRP3-dependent maturation of IL-1β (57, 58). Goitrin, an alkaloid derived from Radix Isatis, has been shown to exert protective effects against LPS-induced septic shock by inhibiting the caspase-11-mediated non-canonical inflammasome pathway (59, 60).

### 2.3 Downstream effector molecules

Activation of the NLRP3 inflammasome leads to the self-cleavage and activation of caspase-1, which in turn drives the maturation and secretion of IL-1β and IL-18 and directly induces pyroptosis. Pyroptosis is primarily mediated by caspase-1-dependent cleavage of gasdermin D (GSDMD), generating an N-terminal fragment that forms pores in the plasma membrane, resulting in the release of cellular contents and amplification of the inflammatory response (24, 61).

Substantial evidence indicates that aberrant activation of NLRP3 inflammasome effector molecules contributes to the pathogenesis of various inflammatory diseases. In models of sepsis, metabolic disorders, cardiovascular diseases, and liver injury, the NLRP3 inflammasome activation is consistently associated with increased caspase-1 activity and elevated secretion of IL-1β and IL-18 (62–65). Furthermore, NLRP3-induced pyroptosis via GSDMD leads to the release of additional inflammatory mediators, exacerbating both local and systemic inflammation (66).

The NLRP3 inflammasome plays a multifaceted role in immune responses and inflammatory dysregulation by promoting the release of various cytokines and regulating multiple forms of cell death, including apoptosis, necroptosis, and ferroptosis (67). Among its downstream effectors, caspase-1, IL-1β, IL-18, and the pyroptotic pathway represent the most proximal and direct targets for therapeutic intervention, as they are the primary executors of the acute inflammatory cascade following inflammasome activation (68, 69).

### 2.4 Autophagy and its interplay with the NLRP3 inflammasome

Autophagy, a key homeostatic process, inhibits NLRP3 inflammasome activity by degrading damaged organelles, abnormal proteins, and inflammation-related molecules. p62/SQSTM1-dependent selective autophagy targets and degrades the NLRP3 inflammasome, thereby attenuating inflammatory responses (70, 71). Specifically, K63-polyubiquitinated NLRP3 is recognized by p62 and delivered to autolysosomes for degradation (72, 73). Autophagy-related proteins also participate in this process (74).

Impaired autophagy promotes NLRP3 hyperactivation by allowing accumulation of damaged mitochondria, ROS, and mtDNA, which drive inflammasome assembly (75–77). Lysosomal dysfunction further impairs autophagic flux, preventing NLRP3 clearance and sustaining inflammation (78).In clinical contexts—including sepsis-related immune imbalance, diabetic complications, Alzheimer’s disease, and Parkinson’s disease—impaired autophagy is associated with aberrant NLRP3 inflammasome activation and excessive cytokine release, aggravating tissue damage and disease progression (79, 80). Notably, certain proteins such as Notch1 and HDAC6 are upregulated under autophagy-deficient conditions and further promote NLRP3 inflammasome activation and chronic inflammation (81). Moreover, several drugs or pathogens can inhibit autophagy, indirectly leading to dysregulated NLRP3 inflammasome activation (82, 83).

## 3 Role of the NLRP3 inflammasome in immune imbalance during sepsis

### 3.1 Role in the hyperactivated immune phase

The hyperinflammatory phase, also known as the cytokine storm, is triggered by PAMPs and DAMPs released during infection. This phase is characterized by excessive activation of innate immune cells, leading to overproduction of pro-inflammatory cytokines such as TNF-α, IL-1, and IL-6 (84). Upon pathogen invasion, PAMPs and DAMPs are recognized by the complement system and pattern recognition receptors (PRRs), initiating innate immune responses. Increased expression of vascular endothelial adhesion molecules and widening of endothelial intercellular spaces facilitate immune cell adhesion and extravasation to the infection site (85). Simultaneously, professional antigen-presenting cells present antigens via major histocompatibility complex (MHC) class II molecules to initiate adaptive immunity, coordinating the clearance of invading pathogens (86).

During the hyperinflammatory phase of sepsis, the NLRP3 inflammasome promotes caspase-1 activation, which in turn catalyzes the cleavage and secretion of proinflammatory cytokines IL-1α, HMGB1 and IL-33 (87). In the lungs, the NLRP3 inflammasome promotes macrophage polarization and inflammatory cytokine release, leading to alveolar epithelial pyroptosis and increased permeability, thereby aggravating lung injury (88, 89). In the liver, NLRP3-mediated pyroptosis contributes to hepatocyte death and inflammatory infiltration, while in the kidneys, it promotes acute kidney injury, collectively worsening sepsis progression (90, 91).

Beyond directly fueling the cytokine storm, the NLRP3 inflammasome interacts with metabolic reprogramming and oxidative stress pathways to further amplify inflammation. For instance, elevated ROS during sepsis directly activates the NLRP3 inflammasome, forming a positive feedback loop that sustains inflammatory cascades (92). This notion is further supported by the ability of ROS scavengers such as MitoTEMPO and N-acetyl-L-cysteine to suppress NLRP3 inflammasome activation and mitigate inflammatory injury (93, 94).Lipid metabolism disorders and mitochondrial dysfunction can also enhance NLRP3 activity by modulating its post-translational modifications and inflammasome assembly, resulting in uncontrolled inflammation (95). At the molecular level, dysregulated expression of signaling molecules—such as p38 MAPK and the GDF3–SMAD2/3 axis—can influence the assembly and activation of NLRP3, shaping the intensity and duration of the inflammatory response (96, 97).

From a translational perspective, NLRP3 inhibitors (e.g., MCC950 and dipyridamole) have shown promising anti-inflammatory and organ-protective effects in animal models, significantly reducing inflammatory cytokine levels, attenuating tissue damage, and improving survival (14, 98).In summary, the pivotal role of the NLRP3 inflammasome in the hyperactivated immune phase of sepsis provides a strong rationale for developing novel anti-inflammatory therapies and offers potential targets for preventing multiple organ dysfunction syndrome (MODS).

### 3.2 Role in the immunoparalysis phase

Although inflammasome activation is critical for initiating antimicrobial responses, persistent stimulation can drive immunoparalysis. This phase is characterized by lymphocyte apoptosis, reduced capacity to produce proinflammatory cytokines, excessive expansion of regulatory cells, and abundant secretion of anti−inflammatory mediators (99). Reduced HLA−DR expression on CD14^+^CD16^-^ classical monocytes is a hallmark of sepsis−induced immunosuppression. Lymphocyte apoptosis affects multiple immune subsets, including CD4^+^ and CD8^+^ T cells, B cells, NK cells and dendritic cells. T cell exhaustion is also prominent during sepsis (100).

As sepsis progresses, patients often transition from early hyperinflammation to a later immunosuppressive state, referred to as the immunoparalysis phase. Although strong activation of the NLRP3 inflammasome in early sepsis promotes inflammation and pyroptosis, its sustained activation can lead to subsequent downregulation and impaired immune cell function (101). This shift partly reflects a negative feedback mechanism aimed at preventing continuous tissue damage. However, the decline in NLRP3 inflammasome expression and inflammasome activity weakens both innate and adaptive immunity, reducing the capacity of immune cells—such as macrophages, dendritic cells, and T cells—to recognize and eliminate new pathogens. Several mechanisms underlie this downregulation. First, negative feedback regulation mediated by anti-inflammatory cytokines suppresses NLRP3 expression and inflammasome assembly to limit excessive tissue damage (102). Second, endotoxin tolerance, a state of hyporesponsiveness to repeated LPS stimulation, is associated with reduced NLRP3 transcription and diminished inflammasome activity in monocytes and macrophages (103). Third, metabolic reprogramming involving mTOR inhibition and AMPK activation during the late phase of sepsis shifts cellular metabolism toward oxidative phosphorylation, which is linked to decreased NLRP3 inflammasome activation (104).

A key feature of the immunoparalysis phase is an increased risk of secondary infections. Prolonged NLRP3 inflammasome activation and eventual exhaustion can promote immune tolerance. For example, some studies have linked reduced NLRP3 inflammasome activity to increased numbers of myeloid-derived suppressor cells (MDSCs), impaired antigen presentation, and T cell anergy, collectively hampering the clearance of pathogens and malignant cells (10).

Notably, patients exhibit considerable heterogeneity in their immunoparalysis phenotype. Transcriptomic analyses of NLRP3-related gene expression in septic patients have identified distinct subtypes: one with rapid inflammatory resolution and another with persistent hyperinflammation. The latter group displays not only sustained inflammation but also marked immunosuppression and higher 28-day mortality (101), underscoring the close relationship between NLRP3 dynamics and immune imbalance in sepsis.

### 3.3 Dynamic regulation and mechanisms of imbalance

**Figure 2:**
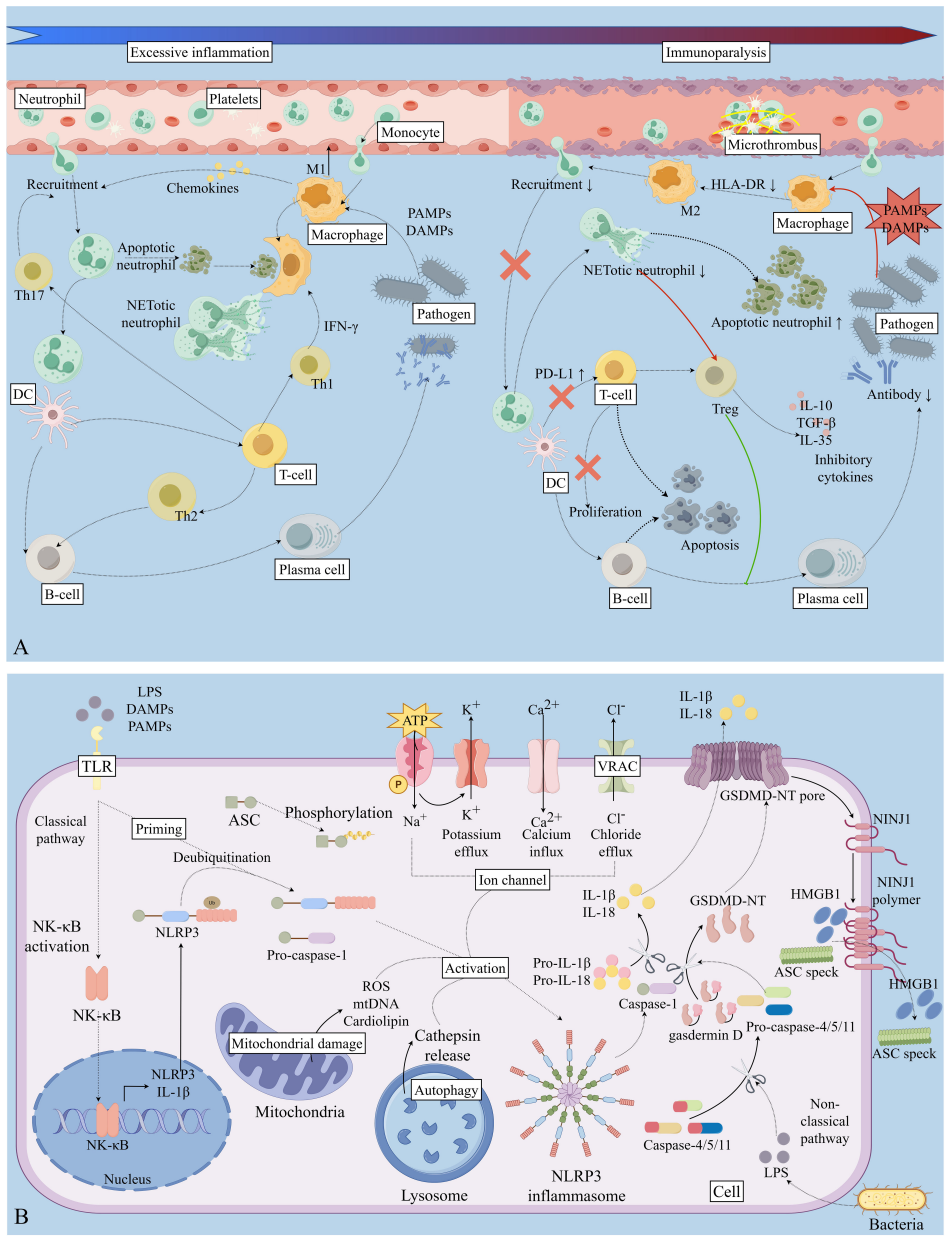
Activation of NLRP3 inflammasome and immune response. **(A)** The progression of the body to an immunosuppressive or dysfunctional state under continuous immune stimulation. **(B)** The mechanism of NLRP3 inflammasome activation. The image was generated using Figdraw. LPS, Lipopolysaccharide; DAMPs, Damage-Associated Molecular Patterns; PAMPs, Pathogen-Associated Molecular Patterns; HLA-DR, Human Leukocyte Antigen - DR isotype; IFN-γ, Interferon-gamma; IL-10, Interleukin-10; IL-35, Interleukin-35; IL-1β, Interleukin-1β; NET, Neutrophil Extracellular Trap; PAMPs, Pathogen-Associated Molecular Patterns; PD-L1, Programmed Death-Ligand 1; TGF-β, Transforming Growth Factor-β; Th1, T helper 1 cell; Th2, T helper 2 cell; Th17, T helper 17 cell; Treg, Regulatory T cell; ASC, Apoptosis-associated speck-like protein containing a CARD; NF-κB, Nuclear factor kappa-light-chain-enhancer of activated B cells; DC, Dendritic Cell; GSDMD-NT, Gasdermin D N-terminal domain; NINJ1, nerve injury induced protein 1; HMGB1, High Mobility Group Box 1.

The immune response in sepsis reflects a complex equilibrium between activation and suppression, in which the NLRP3 inflammasome serves as a central inflammatory regulator and a key factor in the loss of immune homeostasis (11). Abnormal activation of NLRP3 inflammasome is closely associated with multiple sepsis-related organ injuries (**Figure 2**), and its dysregulation represents a core mechanism driving disease progression (10, 14).

At the molecular level, NLRP3 inflammasome activation is modulated by multiple signaling events, including ROS, metabolic reprogramming, post-translational modifications, and endoplasmic reticulum stress (105). Excessive ROS production not only causes direct cellular damage but also promotes NLRP3 inflammasome assembly and activation, thereby amplifying the inflammatory response (92). In addition, metabolic reprogramming in immune cells—such as altered energy metabolism in macrophages—is closely linked to NLRP3 inflammasome activation. Metabolic imbalance can further exacerbate inflammation, establishing a vicious cycle (106). Protein modifications also influence inflammasome dynamics by regulating NLRP3 stability and activation thresholds. For example, glucocorticoid-Induced TNFR-related protein (GITR) enhances inflammatory responses and disrupts immune balance by modulating the ubiquitination and acetylation status of NLRP3 (95).

Notably, NLRP3 inflammasome activation exerts a “double-edged sword” effect: appropriate activation aids in pathogen clearance and initiates immune defense, whereas excessive or sustained activation leads to tissue damage and immune exhaustion, compromises host defense against secondary infections, and promotes the development of immunoparalysis (10).Therefore, precise regulation of the NLRP3 inflammasome represents a promising strategy for restoring immune balance and improving outcomes in sepsis.

## 4 Interaction between the NLRP3 inflammasome and immune cells in sepsis

### 4.1 Monocyte–macrophage system

Activation of the NLRP3 inflammasome in the monocyte–macrophage system plays a critical role in the immune paralysis–hyperactivation imbalance during sepsis. Studies indicate that macrophages in early sepsis are influenced by various molecular signals. For example, elevated lysophosphatidylcholine (LPC) induces GITR expression on macrophages, which enhances NLRP3 inflammasome activation and macrophage pyroptosis by modulating post-translational modifications of NLRP3, thereby exacerbating systemic inflammatory injury (49). In addition, the lysosome-anchored Ragulator complex promotes NLRP3 inflammasome activation through interactions with NLRP3 and HDAC6. Deletion of Lamtor1 significantly attenuates NLRP3-dependent inflammatory responses in macrophages (107).

Inhibition of NLRP3 inflammasome activation alleviates inflammation and organ damage. Hederagenin, for instance, suppresses the NLRP3 inflammasome and NF-κB signaling, reduces M1 macrophage polarization and proinflammatory cytokine release, and consequently improves lung injury and survival in septic mice (108). Furthermore, long-term follow-up studies suggest that sepsis can induce lasting reprogramming of the inflammatory state in monocytes, characterized by sustained overexpression of NLRP3 and related signaling molecules. This altered state may persist for months or even years after hospital discharge, indicating a role for NLRP3 inflammasome in maintaining immune dysfunction and immunoparalysis (109).

NLRP3 inflammasome activation involves multiple signaling cascades. For example, TLR4 and the P2X7 receptor act synergistically to induce NLRP3 inflammasome assembly, promoting the maturation and release of IL-1β and other proinflammatory cytokines, which amplifies the inflammatory cascade (110, 111). Oxidative stress and ROS generation not only act as upstream signals for NLRP3 inflammasome activation but also facilitate the formation and translocation of the TXNIP–NLRP3 complex, thereby propagating inflammatory signaling among monocytes and macrophages and aggravating organ injury (112).

### 4.2 Neutrophils and lymphocytes: effects of NLRP3-mediated pyroptosis on immune cell number and function

Activation of the NLRP3 inflammasome in sepsis and other inflammatory conditions directly influences the number and function of neutrophils and lymphocytes, thereby modulating immune balance. Upon activation, NLRP3 triggers pyroptosis, leading to dynamic changes in immune cell populations. Studies indicate that NLRP3 activation promotes neutrophil recruitment and infiltration, enhancing their accumulation and bactericidal capacity at inflammatory sites. For example, in models of acute inflammation and sepsis, NLRP3 activation facilitates neutrophil migration and accumulation in peripheral blood and tissues, accompanied by the release of IL-1β and IL-18, which exacerbates local or systemic inflammation (113–115). Moreover, NLRP3-mediated pyroptosis not only reduces immune cell numbers but also amplifies inflammation through the release of intracellular contents that further activate additional immune cells (116).

NLRP3 inflammasome activation also plays an important regulatory role in lymphocytes. It has been shown to participate in B-cell proliferation, antibody secretion, and the release of inflammation-related cytokines, as well as in shaping T-cell subset distribution and function, thereby influencing adaptive immune responses (117). Studies have shown that excessive NLRP3 activation increases lymphocyte apoptosis (118), serving as a key contributor to the development of immunoparalysis. Immunoparalysis is characterized by reduced peripheral lymphocyte counts and impaired immune function, which represent the fundamental mechanisms underlying secondary infections and multiple organ dysfunction in late-stage sepsis (10). Furthermore, NLRP3-mediated pyroptosis can damage lymphoid organs (e.g., spleen and lymph nodes), exacerbating lymphocyte depletion and dysfunction (119).

The regulatory role of the NLRP3 inflammasome is dual-faced. In early inflammation, appropriate NLRP3 inflammasome activation aids in the effective recruitment and functional activation of neutrophils and lymphocytes, enhancing pathogen clearance. However, in later stages or under overactivation, it may lead to excessive immune cell depletion and functional suppression, inducing immunoparalysis and tissue injury (92). Therefore, precise modulation of the NLRP3 inflammasome and its mediated pyroptotic process may offer new theoretical insights and therapeutic targets for restoring immune balance in sepsis and other inflammatory diseases.

### 4.3 Intercellular signaling networks: regulatory roles of cytokines, chemokines, and extracellular vesicles

The regulatory role of the NLRP3 inflammasome in the immune paralysis–hyperactivation imbalance during sepsis involves complex intercellular signaling networks. Key mechanisms include the interplay and feedback regulation of inflammatory cytokines, chemokines, and extracellular vesicles (EVs) among immune cells. Upon NLRP3 inflammasome activation, the release of IL-1β and IL-18 rapidly activates neighboring immune cells, triggering a cascade that influences immune cell chemotaxis, activation, and functional polarization. For instance, in sepsis-associated acute lung injury and multiple organ dysfunction, NLRP3-mediated secretion of IL-1β and IL-18 promotes the recruitment and activation of neutrophils and monocytes/macrophages, amplifying local and systemic inflammation (120).

Chemokines such as CXCL1 and lipocalin-2 (LCN2) are also upregulated following NLRP3 inflammasome activation, further facilitating immune cell migration and tissue infiltration. In the liver, for example, metabolic stress combined with alcohol exposure enhances CXCL1 production in hepatocytes via the NLRP3–IL-1β axis, recruiting more neutrophils and establishing a feedforward inflammatory loop (121). This process contributes to the development of immune imbalance and immunoparalysis.

Extracellular vesicles serve as essential mediators of intercellular communication in NLRP3-regulated immune networks. EVs derived from various cells—including macrophages, cardiomyocytes, and tumor cells—carry bioactive molecules such as miRNAs, proteins, and cytokines, which can be transferred to recipient cells to modulate NLRP3 inflammasome activity. In severe acute pancreatitis, plasma exosomes enriched in miR-155-5p are delivered to intestinal epithelial cells, activating NLRP3-mediated pyroptosis and disrupting intestinal barrier integrity (122). Similarly, tumor-derived exosomes promote IL-1β secretion and reshape the tumor microenvironment by activating NLRP3 inflammasome in macrophages (123).

Notably, exosomes can also suppress NLRP3 inflammasome activation under certain conditions. For example, exosomes from human amniotic stem cells inhibit NLRP3 inflammasome activation in monocytes via ATP-adenosine conversion and A2a receptor signaling, thereby exerting anti-inflammatory effects (124). Natural exosome-like nanoparticles derived from traditional Chinese medicine have also been shown to alleviate inflammatory conditions such as diabetic cardiomyopathy by modulating the NLRP3 inflammasome and its downstream signaling (125).

In the cardiovascular system, NLRP3 inflammasome activation facilitates the spread and amplification of inflammatory signals through direct intercellular communication and EV-mediated transfer. Membrane nanotubes (MNTs), for instance, enable direct signaling from cardiomyocytes to cardiac fibroblasts, propagating inflammasome activation and promoting pyroptosis related cardiac injury (126). Moreover, cytokine stimulation (e.g., by TNF-α) enhances NLRP3-dependent EV release from cardiomyocytes, influencing inflammatory responses in surrounding cells (127).

In summary, the NLRP3 inflammasome acts through a multi-layered network of cytokines, chemokines, and extracellular vesicles to regulate immune cell recruitment, activation, and functional states. This network not only drives early hyperinflammation in sepsis but also sets the stage for subsequent immune-paralysis, representing a critical node in the maintenance of immune balance and tissue homeostasis. A deeper understanding of these signaling mechanisms will provide a theoretical foundation for precision interventions in sepsis and related inflammatory diseases.

## 5 Regulatory factors of the NLRP3 inflammasome and targeted intervention strategies

### 5.1 Endogenous regulatory factors: microRNAs and long non-coding RNAs in NLRP3 regulation

**Figure 3:**
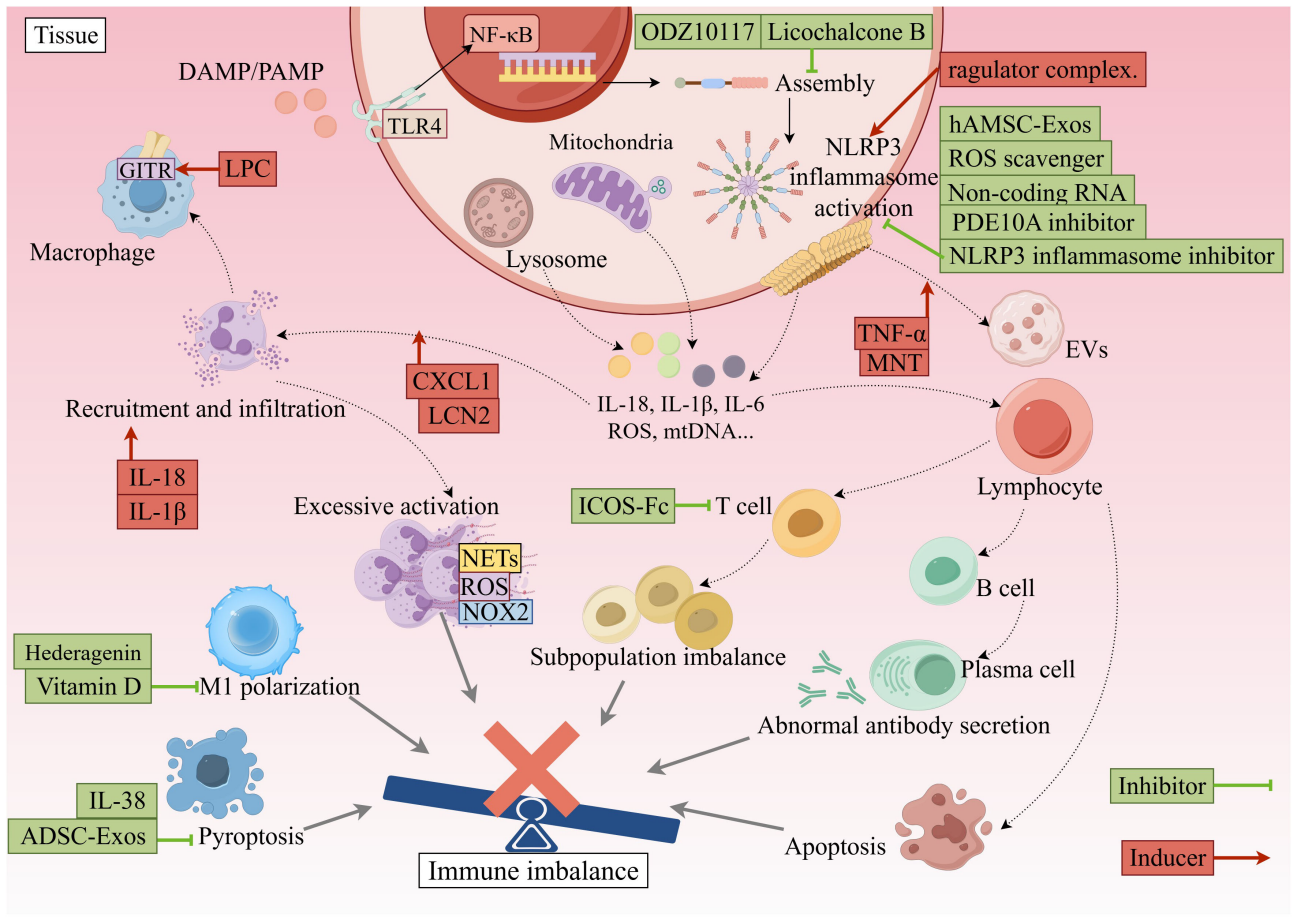
Inhibitors and promoters and their targets of action. This figure shows the key targets and signaling pathways of the immune system, including the TLR4/NF-κB pathway, NLRP3 inflammasome activation, cytokine release, immune cell interactions, and related therapeutic strategies. Red boxes indicate inducers and green boxes indicate inhibitors. The image was generated using Figdraw. ADSC, Adipose-Derived Stem Cell; B cell, B lymphocyte; CXCL1, Chemokine (C-X-C motif) ligand 1; DC, Dendritic Cell; EVs, Extracellular Vesicles; GITR, Glucocorticoid-Induced TNFR-Related protein; ICOS, Inducible T-cell COStimulator; IL-18, Interleukin-18; IL-6, Interleukin-6; LCN2, Lipocalin 2; LPG, Lipophosphoglycan; mtDNA, Mitochondrial DNA; NF-κB, Nuclear Factor kappa-light-chain-enhancer of activated B cells; NLRP3, NLR Family Pyrin Domain Containing 3; NOX2, NADPH Oxidase 2; PDE10A, Phosphodiesterase 10A; ROS, Reactive Oxygen Species; TLR4, Toll-like Receptor 4; IFN-α, Interferon-alpha; NETs, Neutrophil Extracellular Traps.

Endogenous molecules such as microRNAs (miRNAs) and long non-coding RNAs (lncRNAs) function as important epigenetic regulators that exert multi-level control over the expression and activity of the NLRP3 inflammasome (**Figure 3**). Studies have shown that miRNAs can directly or indirectly modulate inflammasome activation by targeting mRNAs of NLRP3 or its upstream signaling components. For instance, miR-369-3p downregulates BRCC3 expression, thereby reducing NLRP3 inflammasome assembly and activation, and subsequently suppressing caspase-1 activity and pro-inflammatory cytokine secretion (128). Similarly, hsa-miR-4282 has been found to inhibit NLRP3 inflammasome assembly and pyroptosis by blocking the NF-κB/NLRP3 pathway, exerting anti-inflammatory and protective effects in an osteoarthritis model (129).

LncRNAs, on the other hand, can influence miRNA expression or function, or directly interact with NLRP3-related proteins to modulate inflammatory responses. Some lncRNAs regulate not only NLRP3 expression itself but also upstream signaling pathways such as TLR4 and NF-κB, thereby contributing to the development of inflammatory diseases like atherosclerosis (130). Additionally, other non-coding RNAs, including circular RNAs (circRNAs), are known to act as miRNA “sponges,” indirectly regulating NLRP3 expression and activation.

Through their multi-level and multi-target regulatory mechanisms, non-coding RNAs participate in the activation, assembly, and downstream signaling of the NLRP3 inflammasome, influencing immune responses and pathological processes in sepsis and other inflammatory diseases. Research in this area not only deepens our understanding of NLRP3 regulation but also provides a theoretical basis and potential targets for developing more precise molecular therapies (131).

### 5.2 Drugs and small-molecule inhibitors

Research on drugs and small-molecule inhibitors targeting the NLRP3 inflammasome has advanced considerably in recent years (see **Figure 3** for an overview). MCC950, a selective NLRP3 inhibitor, effectively suppresses NLRP3 activation and reduces the release of downstream inflammatory factors, thereby attenuating inflammatory responses and tissue injury (132). In experimental models of bacterial infection and toxin-induced sepsis, MCC950 has been shown to prevent NLRP3-mediated inflammation and mortality, highlighting its potential for clinical translation (133). However, no NLRP3 inhibitor has yet been approved for clinical use, and further studies are needed to evaluate the metabolism, safety, and long-term risks of MCC950 (134).

Beyond MCC950, researchers have explored various novel NLRP3 inflammasome inhibitors and delivery strategies. For example, a lipid nanoparticle-based co-delivery system for MCC950 and disulfiram has demonstrated enhanced suppression of NLRP3 inflammasome activation, along with reduced levels of active caspase-1 and IL-1β, suggesting that nanocarrier systems may help overcome limitations in stability, toxicity, and dosing associated with small-molecule inhibitors (135). In various organ disease models, induction of autophagy significantly reduces NLRP3 activation and inflammatory injury (136). Pharmacological or natural activators of autophagy—including metformin, rapamycin, and kakonein—have demonstrated anti-inflammatory and tissue-protective effects by promoting NLRP3 inflammasome degradation (137, 138). Other NLRP3 inflammasome inhibitors, such as disulfiram, clioquinol, and the natural product tabersonine and its derivatives, have also shown promising NLRP3-inhibitory and anti-inflammatory effects in animal models of sepsis and related inflammatory diseases (139–141). Additionally, combination therapies and multi-target inhibition strategies offer new directions for treating sepsis and its complications (134, 135).

In summary, MCC950 and other specific NLRP3 inflammasome inhibitors provide valuable molecular tools and potential translational avenues for rebalancing the immune paralysis–hyperactivation dichotomy in sepsis. However, their clinical application will require multidisciplinary collaboration and further systematic investigation.

### 5.3 Immunomodulators and combination therapy strategies

Sepsis involves a complex immune imbalance, characterized by both hyperinflammation and immunoparalysis. The NLRP3 inflammasome contributes to this process by amplifying inflammatory signaling and inducing pyroptosis, with its dysregulated activation closely linked to disease progression. While NLRP3 inflammasome inhibitors have been shown in animal models to mitigate inflammation and organ damage (142), their clinical translation as monotherapy is hampered by limited efficacy and potential immunosuppression, underscoring the need for combination strategies with immunomodulators to achieve a more balanced therapeutic outcome.

Combination approaches leverage synergistic multi-target and multi-pathway effects. Immunomodulators—such as IL-38, ICOS-Fc, fat-soluble vitamins, and adipose-derived stem cell exosomes—can alleviate hyperinflammation and immunoparalysis by suppressing inflammatory cytokine release, promoting macrophage polarization toward an anti-inflammatory phenotype, and reducing pyroptosis and oxidative stress (143–145).

Currently, clinical evidence for combining immunomodulators with NLRP3 inflammasome inhibitors remains limited, with most studies conducted in animal or *in vitro* models. Nevertheless, several reports indicate that such combinations exert synergistic effects, improving inflammatory responses, reducing organ injury, and enhancing survival. For example, natural compounds like betulinic acid exhibit immunomodulatory activity by inhibiting the NLRP3 inflammasome and modulating macrophage function (146). Future efforts should focus on elucidating the underlying mechanisms, advancing clinical trials, and optimizing combination regimens to enable personalized and stratified treatment for improved patient outcomes.

## 6 NLRP3 inflammasome and advances in clinical translation of sepsis

### 6.1 Development of clinical biomarkers

The NLRP3 inflammasome and its downstream effectors are increasingly recognized as important biomarkers for the diagnosis and prognostic assessment of sepsis. Clinical studies have shown that patients with serum NLRP3 levels above a specific threshold (e.g., 147.72 pg/mL) exhibit significantly higher 30-day mortality, suggesting the potential of NLRP3 for early identification of high-risk individuals and stratification of poor outcomes (147). Current methods for detecting NLRP3 and related molecules—such as enzyme-linked immunosorbent assay (ELISA), flow cytometry, and molecular biology techniques—enable quantitative analysis in serum, peripheral blood mononuclear cells, and other samples (148). Furthermore, the NLRP3 inflammasome provides a foundation for precision medicine and personalized therapy. Integrating artificial intelligence with multi-omics data, multidimensional analysis of NLRP3 inflammasome and its downstream factors may facilitate more accurate immune subtyping and risk prediction models for sepsis, thereby supporting clinical decision-making (149, 150). However, the clinical application of NLRP3 inflammasome and its associated components as biomarkers still faces challenges, including standardization of detection methods and validation of specificity and sensitivity. Future large-scale, multicenter clinical studies are needed to refine detection techniques and evaluation systems, ultimately promoting their translation into the clinical management of sepsis.

### 6.2 Preclinical and clinical trials targeting NLRP3

In recent years, multiple approved drugs and novel molecules targeting the NLRP3 inflammasome have demonstrated favorable efficacy and safety in preliminary studies. Compounds such as melatonin, ulinastatin, irisin, furazolidone, and ginsenoside Rg1 have been shown to effectively inhibit NLRP3 activation in cellular and animal models, suggesting their potential as novel therapeutic candidates for sepsis (151). Disulfiram has also been reported to suppress NLRP3 inflammasome activation and pyroptosis, exhibiting significant anti-inflammatory effects in murine models of peritonitis and gouty inflammation (139).

In animal studies, PDE10A inhibitors such as MP-10 and TP-10 suppressed NLRP3 activation and pyroptosis by blocking ASC speck formation and reducing levels of caspase-1, gasdermin D, and IL-1β (152). Notably, MP-10 has already shown a good safety profile in clinical trials, supporting its potential translation to sepsis and other inflammatory diseases.

Regarding clinical translation, direct or indirect NLRP3 inflammasome inhibitors—including IL-1β and IL-18 antagonists—have entered early-stage clinical trials (10). Dipyridamole, an FDA-approved agent, has been found to mitigate sepsis-associated acute lung injury by inhibiting mtROS release and disrupting the NLRP3–NEK7 interaction, highlighting its translational relevance (14). Several natural products and herbal extracts have also been shown to attenuate sepsis-related organ injury through multi-pathway inhibition of the NLRP3 inflammasome (11). For example, nordalbergin, a coumarin derived from Dalbergia sissoo, reduced serum aspartate aminotransferase (AST), alanine aminotransferase (ALT), and blood urea nitrogen (BUN) levels, attenuated inflammatory cell infiltration, and inhibited NLRP3 inflammasome activation by suppressing ROS production and caspase-1 maturation in animal model (153). The probiotic Saccharomyces boulardii suppressed TLR4/NF-κB/NLRP3 signaling, reduced hepatic necrosis, and preserved epithelial barrier integrity in septic rats (154). Other agents targeting diverse pathways have also shown efficacy in animal models of sepsis. The tyrosine kinase inhibitor sunitinib, administered at low doses in septic rats, reduced proinflammatory cytokines, suppressed NLRP3 inflammasome pathway gene expression, attenuated histopathological changes in multiple organs, and significantly improved survival rates (155, 156). Acitretin, a retinoid drug repurposed from dermatology, attenuated acute lung injury, reduced mortality, and inhibited NLRP3 inflammasome activation in septic mice (157).

Overall, NLRP3-targeting agents generally exhibit promising anti-inflammatory and organ-protective effects in animal models. However, large-scale clinical data remain scarce, and the long-term safety and immunomodulatory consequences of these treatments require further investigation.

### 6.3 Personalized therapy and precision medicine

Activation of the NLRP3 inflammasome not only promotes inflammatory responses and organ injury during the hyperinflammatory phase of sepsis but may also contribute to impaired host defense in the immunoparalytic stage, thereby influencing patient outcomes. Stratifying patients based on NLRP3-related molecular features could thus provide a basis for precise clinical intervention (147).Although studies in immune−mediated and rheumatic diseases have suggested a correlation between serum NLRP3 levels and long−term prognosis, large−scale clinical data validating this association in sepsis patients remain limited (158). Nonetheless, given the distinctive pathophysiological characteristics and dynamic nature of the immune response in sepsis, this approach holds considerable promise for future clinical application.

Studies have indicated that NLRP3 inflammasome expression exhibits high sensitivity and specificity and correlates closely with clinical severity scores such as the n-SOFA, offering a molecular basis for early identification and risk assessment of neonatal sepsis (159). Furthermore, research into the molecular mechanisms of NLRP3 inflammasome activation has revealed that metabolic reprogramming, oxidative stress, and post-translational modifications regulate its activity. These features not only reflect interpatient heterogeneity but also support the selection of individualized therapeutic targets (95, 106).

Several drugs and molecular inhibitors have been shown to specifically inhibit NLRP3 activation. For example, dipyridamole significantly improved outcomes in an animal model of sepsis-associated acute lung injury, suggesting that patients with high NLRP3 expression or activity may benefit from such inhibitors (14). Other agents, including MCC950 and Licochalcone B, have also demonstrated potential to suppress NLRP3 activation and attenuate organ injury in various models, expanding the options for targeted therapy (160).

Notably, the activation state and regulatory mechanisms of the NLRP3 inflammasome vary considerably among patients. Specific signaling pathways or proteins have been found to play prominent roles in regulating NLRP3 inflammasome in certain individuals, highlighting opportunities for molecular subtyping and tailored interventions (90, 95, 161). In the future, integrated multi-omics analyses (e.g., genomic, transcriptomic, and proteomic data) of NLRP3-associated molecular profiles may enable precise stratification of sepsis patients, guide personalized drug selection and timing of interventions, and ultimately improve therapeutic efficacy while minimizing adverse effects.

## 7 Future perspectives and challenges

### 7.1 Hotspots and frontiers in NLRP3 inflammasome research

Bibliometric analyses indicate that current research on the NLRP3 inflammasome primarily focuses on its molecular activation mechanisms, its role in the pathogenesis of related diseases, and the development of NLRP3-targeting therapeutics (162–164).

In terms of molecular regulation, SUMOylation and ubiquitination play key roles in modulating NLRP3 protein stability and activation. For example, TRIM28 enhances NLRP3 expression by promoting its SUMOylation, whereas the SUMO-specific protease SENP3 suppresses NLRO3 activation through deSUMOylation (53, 54). In addition, Ubc13-mediated K63-linked ubiquitination has been shown to promote NLRP3 activation, while SLC25A3 negatively regulates NLRP3 activity via ubiquitination (165).

In the field of structural biology, the LRR domain of NLRP3 does not function as a uniform inhibitory element but exhibits both positive and negative regulatory effects depending on the activating ligands. The HD1/HD2 “hinge” region and the nucleotide-binding domain perform distinct functions in response to different activators. Furthermore, the PYD and linker regions of NLRP3 can cooperate to mediate partial activation (51). Proteins such as the cell cycle regulator APC10 and β-catenin have also been found to interact directly with NLRP3, regulating its activation and downstream signaling (166, 167).

Cellular metabolic events—including energy metabolism, oxidative stress, mitochondrial DNA release, and fatty acid oxidation—also serve as upstream signals for NLRP3 inflammasome activation, further expanding its regulatory network (168).

The discovery of these novel regulatory mechanisms has facilitated the development of specific small-molecule inhibitors. Compounds such as ODZ10117 and Licochalcone B, for instance, selectively inhibit NLRP3 inflammasome activation by disrupting the NLRP3–NEK7 interaction or binding site (160, 169). Other emerging regulatory molecules, including molecular chaperones, deubiquitinating enzymes, and mitochondrial proteins, have also become potential targets for drug discovery. Moving forward, investigations into novel regulatory mechanisms, structural foundations, and specific NLRP3-targeted agents will continue to shape the forefront of this field.

### 7.2 Challenges in clinical translation

The NLRP3 inflammasome, recognized for its central role in immune dysregulation during sepsis, represents a promising therapeutic target. However, translating NLRP3-related basic research into clinical practice faces several challenges.

First, safety remains a primary concern. Excessive inhibition of the NLRP3 inflammasome may compromise host defense mechanisms, increasing the risk of secondary infections or viral reactivation. Second, specificity is a major issue. Most existing NLRP3 inhibitors lack sufficient target selectivity, potentially interfering with other inflammasomes or off-target cells and leading to broad immunosuppression or unintended effects. Moreover, achieving a therapeutic balance is difficult: excessive suppression may induce immunoparalysis and raise the risk of late-stage infections and mortality, whereas insufficient inhibition fails to control inflammatory responses and improve organ function or outcomes.

Although several NLRP3 inhibitors have demonstrated efficacy in animal models, their long-term safety, pharmacokinetic profiles, and comprehensive effects on the immune system require further evaluation in clinical settings. Additionally, the considerable heterogeneity among sepsis patients—evident in divergent immune states and inflammatory responses—precludes a one-size-fits-all approach. There is an urgent need to develop biomarker-guided, individualized treatment strategies rooted in precision medicine.

### 7.3 Interdisciplinary integration and innovative therapeutic models

The integration of molecular medicine, systems biology, and informatics is advancing NLRP3 inflammasome research in sepsis into a new phase of interdisciplinary collaboration and innovative treatment models. Integrated multi-omics analyses help elucidate the regulatory mechanisms, functional interactions, and roles of NLRP3-related molecules in immune dysregulation, thereby laying the groundwork for novel target discovery. In-depth analysis of clinical data and patient stratification can clarify the complex relationships between NLRP3 activation and various immune phenotypes, organ injury, and clinical outcomes, advancing the field of precision medicine.

Artificial intelligence (AI), particularly machine learning and deep learning algorithms, is becoming a vital tool in NLRP3 inflammasome drug screening and mechanistic studies. For example, several studies have employed bespoke machine learning models to screen large compound libraries for potential NLRP3 inflammasome inhibitors, with subsequent *in vitro* and *in vivo* validation of their efficacy and safety. This approach not only accelerates drug discovery but also provides a robust technical foundation for developing novel anti-inflammatory agents. Furthermore, AI can help predict patient-specific responses to NLRP3-targeted therapies, supporting personalized treatment strategies and improving the success rate of clinical translation.

The impact of interdisciplinary collaboration extends beyond basic research and drug development, showing considerable potential in clinical translation and therapeutic monitoring. For instance, AI-assisted medical image analysis and biomarker mining may enable dynamic tracking of NLRP3 inflammasome activation, allowing early warning and guiding the timing and evaluation of clinical interventions.

## 8 Conclusion

The NLRP3 inflammasome serves as a central regulator in the dysregulated immune response of sepsis, characterized by the oscillation between immunoparalysis and hyperactivation. Its dual role permeates all stages of the disease. Recent basic and clinical research has progressively uncovered the molecular mechanisms by which the NLRP3 inflammasome governs the initiation, propagation, and resolution of inflammatory responses. These advances not only deepen our understanding of sepsis pathophysiology but also provide a rationale for diagnosis, prognosis assessment, and targeted intervention. By sensing diverse exogenous and endogenous danger signals and activating downstream inflammatory cascades, the NLRP3 inflammasome bridges molecular mechanisms with clinical manifestations, standing as an indispensable hub for understanding the immune dynamics in sepsis.

In diagnostics, the expression levels of the NLRP3 inflammasome and its downstream effectors have been closely linked to sepsis severity and outcomes, highlighting their potential as biomarkers for early detection and dynamic monitoring. Concurrently, the development of therapeutics targeting the NLRP3 inflammasome and its associated pathways has gained considerable momentum. Various small-molecule inhibitors, monoclonal antibodies, and nucleic acid-based agents have shown promising intervention potential in preclinical and early clinical studies, offering opportunities to move beyond conventional anti-infective and supportive care toward more precise and personalized treatment.

However, translating NLRP3-focused research into clinical practice faces several challenges. First, the high heterogeneity of sepsis—evident in the substantial interpatient variability in immune status, inflammatory response, and clinical phenotype—necessitates tailored NLRP3 modulation, which will require integration of multi-omics data and large-scale biomarkers. Second, the efficacy and safety profiles of existing NLRP3 inhibitors observed in animal studies and early trials need further validation through large-scale, multicenter clinical trials. Moreover, the physiological role of NLRP3 inflammasome in host defense mandates a cautious approach to targeting it, as excessive suppression may compromise antimicrobial immunity and lead to new complications.

Looking forward, interdisciplinary convergence of immunology, molecular biology, and bioinformatics, coupled with advances in targeted drug delivery and molecular imaging, may position the NLRP3 inflammasome as a cornerstone of precision medicine in sepsis. Collaborative and innovative efforts across fields are expected to revitalize its clinical translation, ultimately shifting sepsis management from empiric therapy to mechanism-based, individualized intervention. Researchers and clinicians should continue to track progress in NLRP3 inflammasome research and explore its practical value in sepsis management, with the ultimate goal of improving patient outcomes through precision therapeutics.

## References

[1] Shappell C Rhee C Klompas M . Update on sepsis epidemiology in the era of COVID-19. Semin Respir Crit Care Med. (2023) 44:173–84. doi: 10.1055/s-0042-1759880. PMID: 36646093

[2] Modugula S Altenbaugh M Ivanova M DuMont T Arshad H . Sepsis epidemiology, definitions, scoring systems, and diagnostic markers. Crit Care Nurs Q. (2025) 48:229–36. doi: 10.1097/CNQ.0000000000000570. PMID: 40423381

[3] Arina P Hofmaenner DA Singer M . Definition and epidemiology of sepsis. Semin Respir Crit Care Med. (2024) 45:461–8. doi: 10.1055/s-0044-1787990. PMID: 38968960

[4] Zhong X Lin R Zhang W Huang S Luo Y Wang D . Epidemiology and clinical features of maternal sepsis: A retrospective study of whole pregnancy period. Medicine. (2022) 101:e30599. doi: 10.1097/MD.0000000000030599. PMID: 36221418 PMC9543042

[5] Rudd KE Johnson SC Agesa KM Shackelford KA Tsoi D Kievlan DR . Global, regional, and national sepsis incidence and mortality, 1990–2017: Analysis for the Global Burden of Disease Study. Lancet. (2020) 395:200–11. doi: 10.1016/S0140-6736(19)32989-7. PMID: 31954465 PMC6970225

[6] Mellhammar L Wollter E Dahlberg J Donovan B Olséen C-J Wiking PO . Estimating sepsis incidence using administrative data and clinical medical record review. JAMA Netw Open. (2023) 6:e2331168. doi: 10.1001/jamanetworkopen.2023.31168. PMID: 37642964 PMC10466163

[7] Rice B Calo S Kamugisha JB Kamara N Chamberlain S Global Emergency Care Investigator Study Group . Emergency care of sepsis in sub-Saharan Africa: Mortality and non-physician clinician management of sepsis in rural Uganda from 2010 to 2019. PloS One. (2022) 17:e0264517. doi: 10.1371/journal.pone.0264517. PMID: 35544466 PMC9094533

[8] Koyner JL . Sepsis and kidney injury. In: Silva Junior GB Ferreiro Fuentes A Nangaku M Remuzzi G Ronco C , editors. Contributions to Nephrology, vol. 199. Basel, Switzerland: S. Karger AG (2021). p. 56–70. doi: 10.1159/000517701, PMID: 34343994

[9] Wang D Qu X Zhang Z Zhou G . New developments in the role of ferroptosis in sepsis-induced cardiomyopathy (review). Mol Med Rep. (2025) 31:1–12. doi: 10.3892/mmr.2025.13483. PMID: 40052561 PMC11904766

[10] Vigneron C Py BF Monneret G Venet F . The double sides of NLRP3 inflammasome activation in sepsis. Clin Sci. (2023) 137:333–51. doi: 10.1042/CS20220556. PMID: 36856019

[11] He W Dong H Wu C Zhong Y Li J . The role of NLRP3 inflammasome in sepsis: A potential therapeutic target. Int Immunopharmacol. (2023) 115:109697. doi: 10.1016/j.intimp.2023.109697. PMID: 37724951

[12] Jang JH Choi E Kim T Yeo HJ Jeon D Kim YS . Navigating the modern landscape of sepsis: Advances in diagnosis and treatment. Int J Mol Sci. (2024) 25:7396. doi: 10.3390/ijms25137396. PMID: 39000503 PMC11242529

[13] Shi X Tan S Tan S . NLRP3 inflammasome in sepsis (review). Mol Med Rep. (2021) 24:514. doi: 10.3892/mmr.2021.12153. PMID: 33982766

[14] Chen X Zheng Y Zhang X Zheng A Huang J Deng G . Harnessing FDA-approved dipyridamole to inhibit NLRP3 inflammasome and improve outcomes of acute lung injury in sepsis. Toxicol Appl Pharmacol. (2025) 500:117383. doi: 10.1016/j.taap.2025.117383. PMID: 40360054

[15] Li J Bai Y Tang Y Wang X Cavagnaro MJ Li L . A 4-benzene-indol derivative alleviates LPS-induced acute lung injury through inhibiting the NLRP3 inflammasome. Front Immunol. (2022) 13:812164. doi: 10.3389/fimmu.2022.812164. PMID: 35222388 PMC8866853

[16] Fu J Wu H . Structural mechanisms of NLRP3 inflammasome assembly and activation. Annu Rev Immunol. (2023) 41:301–16. doi: 10.1146/annurev-immunol-081022-021207. PMID: 36750315 PMC10159982

[17] Xu Q Zhao B Ye Y Li Y Zhang Y Xiong X . Relevant mediators involved in and therapies targeting the inflammatory response induced by activation of the NLRP3 inflammasome in ischemic stroke. J Neuroinflamm. (2021) 18:123. doi: 10.1186/s12974-021-02137-8. PMID: 34059091 PMC8166383

[18] Kim S-K . The mechanism of the NLRP3 inflammasome activation and pathogenic implication in the pathogenesis of gout. J Rheumatic Dis. (2022) 29:140–53. doi: 10.4078/jrd.2022.29.3.140. PMID: 37475970 PMC10324924

[19] Nagar A Rahman T Harton JA . The ASC speck and NLRP3 inflammasome function are spatially and temporally distinct. Front Immunol. (2021) 12:752482. doi: 10.3389/fimmu.2021.752482. PMID: 34745125 PMC8566762

[20] Hara H Tsuchiya K Kawamura I Fang R Hernandez-Cuellar E Shen Y . Phosphorylation of the adaptor ASC acts as a molecular switch that controls the formation of speck-like aggregates and inflammasome activity. Nat Immunol. (2013) 14:1247–55. doi: 10.1038/ni.2749. PMID: 24185614 PMC4813763

[21] Franklin BS Bossaller L De Nardo D Ratter JM Stutz A Engels G . The adaptor ASC has extracellular and “prionoid” activities that propagate inflammation. Nat Immunol. (2014) 15:727–37. doi: 10.1038/ni.2913. PMID: 24952505 PMC4116676

[22] Huang W Wang B Ou Q Zhang X He Y Mao X . ASC-expressing pyroptotic extracellular vesicles alleviate sepsis by protecting B cells. Mol Therapy: J Am Soc Gene Ther. (2024) 32:395–410. doi: 10.1016/j.ymthe.2023.12.008. PMID: 38093517 PMC10861962

[23] Xiao M Zhang P Chen Z Liu X Wei W He Z . Adenosine diphosphate ribosylation factor 6 inhibition protects burn sepsis induced lung injury through preserving vascular integrity and suppressing ASC inflammasome transmission. Burns: J Int Soc For Burn Injuries. (2024) 50:913–23. doi: 10.1016/j.burns.2024.01.009. PMID: 38267288

[24] Guan Y Gu Y Li H Liang B Han C Zhang Y . NLRP3 inflammasome activation mechanism and its role in autoimmune liver disease. Acta Biochim Biophys Sin. (2022) 54:1577–86. doi: 10.3724/abbs.2022137. PMID: 36148948 PMC9828325

[25] Zheng X Zhao D Jin Y Liu Y Liu D . Role of the NLRP3 inflammasome in gynecological disease. Biomedicine Pharmacotherapy. (2023) 166:115393. doi: 10.1016/j.biopha.2023.115393. PMID: 37660654

[26] Ohto U Kamitsukasa Y Ishida H Zhang Z Murakami K Hirama C . Structural basis for the oligomerization-mediated regulation of NLRP3 inflammasome activation. Proc Natl Acad Sci. (2022) 119:e2121353119. doi: 10.1073/pnas.2121353119. PMID: 35254907 PMC8931350

[27] Paik S Kim JK Silwal P Sasakawa C Jo E-K . An update on the regulatory mechanisms of NLRP3 inflammasome activation. Cell Mol Immunol. (2021) 18:1141–60. doi: 10.1038/s41423-021-00670-3. PMID: 33850310 PMC8093260

[28] Paik S Kim JK Shin HJ Park E-J Kim IS Jo E-K . Updated insights into the molecular networks for NLRP3 inflammasome activation. Cell Mol Immunol. (2025) 22:563–96. doi: 10.1038/s41423-025-01284-9. PMID: 40307577 PMC12125403

[29] Bauernfeind FG Horvath G Stutz A Alnemri ES MacDonald K Speert D . Cutting edge: NF-kappaB activating pattern recognition and cytokine receptors license NLRP3 inflammasome activation by regulating NLRP3 expression. J Immunol. (2009) 183:787–91. doi: 10.4049/jimmunol.0901363. PMID: 19570822 PMC2824855

[30] Qi A Liu Y Zhai J Wang Y Li W Wang T . RNF20 deletion causes inflammation in model of sepsis through the NLRP3 activation. Immunopharmacol Immunotoxicol. (2023) 45:469–78. doi: 10.1080/08923973.2023.2170241. PMID: 36650938

[31] He F Cheng Q Li N Shang Y . Carbenoxolone ameliorates allergic airway inflammation through NF-κB/NLRP3 pathway in mice. Biol Pharm Bull. (2022) 45:743–50. doi: 10.1248/bpb.b21-01100. PMID: 35431287

[32] Dadkhah M Sharifi M . The NLRP3 inflammasome: Mechanisms of activation, regulation, and role in diseases. Int Rev Immunol. (2025) 44:98–111. doi: 10.1080/08830185.2024.2415688. PMID: 39402899

[33] Qiu Y Huang Y Chen M Yang Y Li X Zhang W . Mitochondrial DNA in NLRP3 inflammasome activation. Int Immunopharmacol. (2022) 108:108719. doi: 10.1016/j.intimp.2022.108719. PMID: 35349960

[34] Panbhare K Pandey R Chauhan C Sinha A Shukla R Kaundal RK . Role of NLRP3 inflammasome in stroke pathobiology: Current therapeutic avenues and future perspective. ACS Chem Neurosci. (2024) 15:31–55. doi: 10.1021/acschemneuro.3c00536. PMID: 38118278

[35] Xia J Jiang S Dong S Liao Y Zhou Y . The role of post-translational modifications in regulation of NLRP3 inflammasome activation. Int J Mol Sci. (2023) 24:6126. doi: 10.3390/ijms24076126. PMID: 37047097 PMC10093848

[36] Peng X Wang K Zhang C Bao J-P Vlf C Gao J-W . The mitochondrial antioxidant SS-31 attenuated lipopolysaccharide-induced apoptosis and pyroptosis of nucleus pulposus cells via scavenging mitochondrial ROS and maintaining the stability of mitochondrial dynamics. Free Radical Res. (2021) 55:1080–93. doi: 10.1080/10715762.2021.2018426. PMID: 34903138

[37] Meyers AK Wang Z Han W Zhao Q Zabalawi M Duan L . Pyruvate dehydrogenase kinase supports macrophage NLRP3 inflammasome activation during acute inflammation. Cell Rep. (2023) 42:111941. doi: 10.1016/j.celrep.2022.111941. PMID: 36640341 PMC10117036

[38] Fang Y Meng H Wang J . Mechanisms of LPS-induced toxicity in endothelial cells and the protective role of geniposidic acid. Food Chem Toxicol. (2025) 201:115488. doi: 10.1016/j.fct.2025.115488. PMID: 40288513

[39] Karasawa T Komada T Yamada N Aizawa E Mizushina Y Watanabe S . Cryo-sensitive aggregation triggers NLRP3 inflammasome assembly in cryopyrin-associated periodic syndrome. eLife. (2022) 11:e75166. doi: 10.7554/eLife.75166. PMID: 35616535 PMC9177154

[40] Xu Z Chen Z Wu X Zhang L Cao Y Zhou P . Distinct molecular mechanisms underlying potassium efflux for NLRP3 inflammasome activation. Front Immunol. (2020) 11:609441. doi: 10.3389/fimmu.2020.609441. PMID: 33424864 PMC7793832

[41] Pelegrin P . P2X7 receptor and the NLRP3 inflammasome: Partners in crime. Biochem Pharmacol. (2021) 187:114385. doi: 10.1016/j.bcp.2020.114385. PMID: 33359010

[42] Zhang L Tian J Ma L Duan S . Mechanistic insights into severe pulmonary inflammation caused by silica stimulation: The role of macrophage pyroptosis. Ecotoxicology Environ Saf. (2023) 258:114975. doi: 10.1016/j.ecoenv.2023.114975. PMID: 37148754

[43] Lian L Ye X Wang Z Li J Wang J Chen L . Hyperosmotic stress-induced NLRP3 inflammasome activation via the mechanosensitive PIEZO1 channel in dry eye corneal epithelium. Ocular Surface. (2025) 36:106–18. doi: 10.1016/j.jtos.2025.01.005. PMID: 39832672

[44] Sun Y Leng P Song M Li D Guo P Xu X . Piezo1 activates the NLRP3 inflammasome in nucleus pulposus cell-mediated by Ca2+/NF-κB pathway. Int Immunopharmacol. (2020) 85:106681. doi: 10.1016/j.intimp.2020.106681. PMID: 32526681

[45] Zu S Feng Y Zhu C Wu X Zhou R Li G . Acid‐sensing ion channel 1a mediates acid‐induced pyroptosis through calpain‐2/calcineurin pathway in rat articular chondrocytes. Cell Biol Int. (2020) 44:2140–52. doi: 10.1002/cbin.11422. PMID: 32678496

[46] Yang Y Jin S Zhang J Chen W Lu Y Chen J . Acid-sensing ion channel 1a exacerbates renal ischemia–reperfusion injury through the NF-κB/NLRP3 inflammasome pathway. J Mol Med. (2023) 101:877–90. doi: 10.1007/s00109-023-02330-7. PMID: 37246982 PMC10300185

[47] Chae BJ Lee K-S Hwang I Yu J-W . Extracellular acidification augments NLRP3-mediated inflammasome signaling in macrophages. Immune Network. (2023) 23:e23. doi: 10.4110/in.2023.23.e23. PMID: 37416933 PMC10320421

[48] Li C Chen M He X Ouyang D . A mini-review on ion fluxes that regulate NLRP3 inflammasome activation. Acta Biochim Biophys Sin. (2020) 53:131–9. doi: 10.1093/abbs/gmaa155. PMID: 33355638

[49] Xu X Wu X Yue G An Q Lou J Yang X . The role of Nod-like receptor protein 3 inflammasome activated by ion channels in multiple diseases. Mol Cell Biochem. (2023) 478:1397–410. doi: 10.1007/s11010-022-04602-1. PMID: 36378463 PMC10164009

[50] Wang H-H Huang C-R Lin H-C Lin H-A Chen Y-J Tsai K-J . Magnesium-enriched deep-sea water inhibits NLRP3 inflammasome activation and dampens inflammation. Heliyon. (2024) 10:e35136. doi: 10.1016/j.heliyon.2024.e35136. PMID: 39157306 PMC11327587

[51] Rahman Tabassum Nagar A Duffy EB Okuda K Silverman N . NLRP3 sensing of diverse inflammatory stimuli requires distinct structural features. Front Immunol. (2020) 11:1828. doi: 10.3389/fimmu.2020.01828. PMID: 32983094 PMC7479093

[52] Duan Y Wang J Cai J Kelley N He Y . The leucine-rich repeat (LRR) domain of NLRP3 is required for NLRP3 inflammasome activation in macrophages. J Biol Chem. (2022) 298:102717. doi: 10.1016/j.jbc.2022.102717. PMID: 36403854 PMC9763864

[53] Qin Y Li Q Liang W Yan R Tong L Jia M . TRIM28 SUMOylates and stabilizes NLRP3 to facilitate inflammasome activation. Nat Commun. (2021) 12:4794. doi: 10.1038/s41467-021-25033-4. PMID: 34373456 PMC8352945

[54] Shao L Liu Y Wang W Li A Wan P Liu W . SUMO1 SUMOylates and SENP3 deSUMOylates NLRP3 to orchestrate the inflammasome activation. FASEB J. (2020) 34:1497–515. doi: 10.1096/fj.201901653R. PMID: 31914638

[55] Wu J Cai J Tang Y Lu B . The noncanonical inflammasome-induced pyroptosis and septic shock. Semin Immunol. (2023) 70:101844. doi: 10.1016/j.smim.2023.101844. PMID: 37778179

[56] Lee BL Stowe IB Gupta A Kornfeld OS Roose-Girma M Anderson K . Caspase-11 auto-proteolysis is crucial for noncanonical inflammasome activation. J Exp Med. (2018) 215:2279–88. doi: 10.1084/jem.20180589. PMID: 30135078 PMC6122968

[57] Kayagaki N Wong MT Stowe IB Ramani SR Gonzalez LC Akashi-Takamura S . Noncanonical inflammasome activation by intracellular LPS independent of TLR4. Science. (2013) 341:1246–9. doi: 10.1126/science.1240248. PMID: 23887873

[58] Moretti J Jia B Hutchins Z Roy S Yip H Wu J . Caspase-11 interaction with NLRP3 potentiates the noncanonical activation of the NLRP3 inflammasome. Nat Immunol. (2022) 23:705–17. doi: 10.1038/s41590-022-01192-4. PMID: 35487985 PMC9106893

[59] Krause K Franch Arroyo S Ugolini M Kueck T Sullivan TJ Gálvez EJC . Streptococcus pyogenes EVs induce the alternative inflammasome via caspase-4/-5 in human monocytes. EMBO Rep. (2025) 26:4847–85. doi: 10.1038/s44319-025-00558-7. PMID: 40925957 PMC12508482

[60] Ruan D Yang J Luo Q Shi Y Ding L Wang Z . The protective effects of goitrin on LPS-induced septic shock in C57BL/6J mice via caspase-11 non-canonical inflammasome inhibition. Molecules. (2023) 28:2883. doi: 10.3390/molecules28072883. PMID: 37049646 PMC10096381

[61] Huang Y Xu W Zhou R . NLRP3 inflammasome activation and cell death. Cell Mol Immunol. (2021) 18:2114–27. doi: 10.1038/s41423-021-00740-6. PMID: 34321623 PMC8429580

[62] Shi J Guo J Li Z Xu B Miyata M . Importance of NLRP3 inflammasome in abdominal aortic aneurysms. J Atheroscl Thromb. (2021) 28:454–66. doi: 10.5551/jat.RV17048. PMID: 33678767 PMC8193780

[63] Zhan X Li Q Xu G Xiao X Bai Z . The mechanism of NLRP3 inflammasome activation and its pharmacological inhibitors. Front Immunol. (2023) 13:1109938. doi: 10.3389/fimmu.2022.1109938. PMID: 36741414 PMC9889537

[64] Xia Z Zhang C Guo C Song B Hu W Cui Y . Nanoformulation of a carbon monoxide releasing molecule protects against cyclosporin A-induced nephrotoxicity and renal fibrosis via the suppression of the NLRP3 inflammasome mediated TGF-β/Smad pathway. Acta Biomater. (2022) 144:42–53. doi: 10.1016/j.actbio.2022.03.024. PMID: 35304324

[65] Tan Q Ai Q He Y Li F Yu J . P. aeruginosa biofilm activates the NLRP3 inflammasomes *in vitro*. Microb Pathogen. (2022) 164:105379. doi: 10.1016/j.micpath.2021.105379. PMID: 35038547

[66] Bittner ZA Schrader M George SE Amann R . Pyroptosis and its role in SARS-CoV-2 infection. Cells. (2022) 11:1717. doi: 10.3390/cells11101717. PMID: 35626754 PMC9140030

[67] Yu C Chen P Miao L Di G . The role of the NLRP3 inflammasome and programmed cell death in acute liver injury. Int J Mol Sci. (2023) 24:3067. doi: 10.3390/ijms24043067. PMID: 36834481 PMC9959699

[68] Vande Walle L Lamkanfi M . Drugging the NLRP3 inflammasome: from signalling mechanisms to therapeutic targets. Nat Rev Drug Discov. (2024) 23:43–66. doi: 10.1038/s41573-023-00822-2. PMID: 38030687

[69] Diao L Wu Y Jiang X Chen B Zhang W Chen L . Human umbilical cord mesenchymal stem cell-derived exosomes modulate the NLRP3 inflammasome/caspase-1 pathway to repress pyroptosis induced by hypoxia/reoxygenation in cardiac microvascular endothelial cells. Int Heart J. (2024) 65:1107–17. doi: 10.1536/ihj.23-500. PMID: 39477491

[70] Cheng Y Pan X Wang J Li X Yang S Yin R . Fucoidan inhibits NLRP3 inflammasome activation by enhancing p62/SQSTM1-dependent selective autophagy to alleviate atherosclerosis. Oxid Med Cell Longevity. (2020) 2020:1–13. doi: 10.1155/2020/3186306. PMID: 33505579 PMC7812546

[71] Bai R Lang Y Shao J Deng Y Refuhati R Cui L . The role of NLRP3 inflammasome in cerebrovascular diseases pathology and possible therapeutic targets. ASN Neuro. (2021) 13:17590914211018100. doi: 10.1177/17590914211018100. PMID: 34053242 PMC8168029

[72] Zhou Z Zhu X Yin R Liu T Yang S Zhou L . K63 ubiquitin chains target NLRP3 inflammasome for autophagic degradation in ox-LDL-stimulated THP-1 macrophages. Aging. (2020) 12:1747–59. doi: 10.18632/aging.102710. PMID: 32003754 PMC7053591

[73] Wang W Qin Y Song H Wang L Jia M Zhao C . Galectin-9 targets NLRP3 for autophagic degradation to limit inflammation. J Immunol. (2021) 206:2692–9. doi: 10.4049/jimmunol.2001404. PMID: 33963043

[74] Niu Y Zhang Y Zhang W Lu J Chen Y Hao W . Canagliflozin ameliorates NLRP3 inflammasome-mediated inflammation through inhibiting NF-κB signaling and upregulating Bif-1. Front Pharmacol. (2022) 13:820541. doi: 10.3389/fphar.2022.820541. PMID: 35418866 PMC8996145

[75] Song D Tao W Liu F Wu X Bi H Shu J . Lipopolysaccharide promotes NLRP3 inflammasome activation by inhibiting TFEB-mediated autophagy in NRK-52E cells. Mol Immunol. (2023) 163:127–35. doi: 10.1016/j.molimm.2023.09.008. PMID: 37774455

[76] Wang YJ Chen YY Hsiao CM Pan MH Wang BJ Chen YC . Induction of autophagy by pterostilbene contributes to the prevention of renal fibrosis via attenuating NLRP3 inflammasome activation and epithelial-mesenchymal transition. Front Cell Dev Biol. (2020) 8:436. doi: 10.3389/fcell.2020.00436. PMID: 32582712 PMC7283393

[77] Lee S Kim SK Park H Lee YJ Park SH Lee KJ . Contribution of autophagy-Notch1-mediated NLRP3 inflammasome activation to chronic inflammation and fibrosis in keloid fibroblasts. Int J Mol Sci. (2020) 21:8050. doi: 10.3390/ijms21218050. PMID: 33126764 PMC7663397

[78] Li MM Wang X Chen XD Yang HL Xu HS Zhou P . Lysosomal dysfunction is associated with NLRP3 inflammasome activation in chronic unpredictable mild stress-induced depressive mice. Behav Brain Res. (2022) 432:113987. doi: 10.1016/j.bbr.2022.113987. PMID: 35780959

[79] Lu R Zhang L Yang X . Interaction between autophagy and the NLRP3 inflammasome in Alzheimer’s and Parkinson’s disease. Front Aging Neurosci. (2022) 14:1018848. doi: 10.3389/fnagi.2022.1018848. PMID: 36262883 PMC9574200

[80] Li Z Gong C . NLRP3 inflammasome in Alzheimer’s disease: molecular mechanisms and emerging therapies. Front Immunol. (2025) 16:1583886. doi: 10.3389/fimmu.2025.1583886. PMID: 40260242 PMC12009708

[81] Chang P Li H Hu H Li Y Wang T . The role of HDAC6 in autophagy and NLRP3 inflammasome. Front Immunol. (2021) 12:763831. doi: 10.3389/fimmu.2021.763831. PMID: 34777380 PMC8578992

[82] Choi J Jo M Lee E Kim SE Lee DY Choi D . Inhibition of the NLRP3 inflammasome by progesterone is attenuated by abnormal autophagy induction in endometriotic cyst stromal cells: implications for endometriosis. Mol Hum Reprod. (2022) 28:gaac007. doi: 10.1093/molehr/gaac007. PMID: 35333355

[83] Yue C Li J Zhang S Ma R Suo M Chen Y . Activation of the NLRP3-CASP-1 inflammasome is restrained by controlling autophagy during Glaesserella parasuis infection. Veterinary Microbiol. (2024) 295:110160. doi: 10.1016/j.vetmic.2024.110160. PMID: 38964034

[84] van Amstel RBE Bartek B Vlaar APJ Gay E van Vught LA Cremer OL . Temporal transitions of the hyperinflammatory and hypoinflammatory phenotypes in critical illness. Am J Respir Crit Care Med. (2025) 211:347–56. doi: 10.1164/rccm.202406-1241OC. PMID: 39642348 PMC11936145

[85] Bulté D Rigamonti C Romano A Mortellaro A . Inflammasomes: mechanisms of action and involvement in human diseases. Cells. (2023) 12:1766. doi: 10.3390/cells12131766. PMID: 37443800 PMC10340308

[86] Guo L Shen S Rowley JW Tolley ND Jia W Manne BK . Platelet MHC class I mediates CD8+ T-cell suppression during sepsis. Blood. (2021) 138:401–16. doi: 10.1182/blood.2020008958. PMID: 33895821 PMC8343546

[87] Aizawa E Karasawa T Watanabe S Komada T Kimura H Kamata R . GSDME-dependent incomplete pyroptosis permits selective IL-1α release under caspase-1 inhibition. iScience. (2020) 23:101070. doi: 10.1016/j.isci.2020.101070. PMID: 32361594 PMC7200307

[88] Zhou KL He YR Liu YJ Liu YM Xuan LZ Gu ZY . IL-17A/p38 signaling pathway induces alveolar epithelial cell pyroptosis and hyperpermeability in sepsis-induced acute lung injury by activating NLRP3 inflammasome. Adv Biol. (2023) 7:e2300220. doi: 10.1002/adbi.202300220. PMID: 37607110

[89] Zhao Y Wu Y Qu L Hu Y Sun S Yao R . NOD3 reduces sepsis-induced acute lung injury by regulating the activation of NLRP3 inflammasome and the polarization of alveolar macrophages. Inflammation. (2024) 48:2395–406. doi: 10.1007/s10753-024-02197-x. PMID: 39621198

[90] Cao L Wen M Hu Z Jia W Lin J Hu B . Homeodomain-interacting protein kinase 2 regulates NLRP3 inflammasome activation through endoplasmic reticulum stress in septic liver injury. J Int Med Res. (2023) 51:3000605231173272. doi: 10.1177/03000605231173272. PMID: 37190764 PMC10192800

[91] Tanuseputero SA Lin MT Yeh SL Yeh CL . Intravenous arginine administration downregulates NLRP3 inflammasome activity and attenuates acute kidney injury in mice with polymicrobial sepsis. Mediators Inflammation. (2020) 2020:1–11. doi: 10.1155/2020/3201635. PMID: 32454788 PMC7238342

[92] Zhao S Chen F Yin Q Wang D Han W Zhang Y . Reactive oxygen species interact with NLRP3 inflammasomes and are involved in the inflammation of sepsis: from mechanism to treatment of progression. Front Physiol. (2020) 11:571810. doi: 10.3389/fphys.2020.571810. PMID: 33324236 PMC7723971

[93] Chang R Jia H Dong Z Xu Q Liu L Majigsuren Z . Free fatty acids induce apoptosis of mammary epithelial cells of ketotic dairy cows via the Mito-ROS/NLRP3 signaling pathway. J Agric Food Chem. (2023) 71:12645–56. doi: 10.1021/acs.jafc.3c02090. PMID: 37585786

[94] Liu J Huang J Gong B Cheng S Liu Y Chen Y . Polydatin protects against calcium oxalate crystal-induced renal injury through the cytoplasmic/mitochondrial reactive oxygen species-NLRP3 inflammasome pathway. Biomedicine Pharmacotherapy. (2023) 167:115621. doi: 10.1016/j.biopha.2023.115621. PMID: 37793278

[95] Liang S Zhou J Cao C Liu Y Ming S Liu X . GITR exacerbates lysophosphatidylcholine-induced macrophage pyroptosis in sepsis via posttranslational regulation of NLRP3. Cell Mol Immunol. (2024) 21:674–88. doi: 10.1038/s41423-024-01170-w. PMID: 38740925 PMC11214634

[96] Katholnig K Kaltenecker CC Hayakawa H Rosner M Lassnig C Zlabinger GJ . p38α senses environmental stress to control innate immune responses via mechanistic target of rapamycin. J Immunol. (2013) 190:1519–27. doi: 10.4049/jimmunol.1202683. PMID: 23315073 PMC3563859

[97] Jang IH Kruglov V Cholensky SH Smith DM Carey A Bai S . GDF3 promotes adipose tissue macrophage-mediated inflammation via altered chromatin accessibility during aging. United States. (2024). doi: 10.1038/s43587-025-01034-6. PMID: 41398392 PMC12823384

[98] Wang Y Shi Y Zhang X Fu J Chen F . Overexpression of limb bud and heart alleviates sepsis‐induced acute lung injury via inhibiting the NLRP3 inflammasome. Edited by Dr Anupam Jyoti. BioMed Res Int. (2021) 2021:4084371. doi: 10.1155/2021/4084371. PMID: 33553423 PMC7847343

[99] Sun XF Luo WC Huang SQ Zheng YJ Xiao L Zhang ZW . Immune-cell signatures of persistent inflammation, immunosuppression, and catabolism syndrome after sepsis. Med. (2025) 6:100569. doi: 10.1016/j.medj.2024.12.003. PMID: 39824181

[100] Bergmann CB Beckmann N Salyer CE Crisologo PA Nomellini V Caldwell CC . Lymphocyte immunosuppression and dysfunction contributing to persistent inflammation, immunosuppression, and catabolism syndrome (PICS). Shock. (2021) 55:723–41. doi: 10.1097/SHK.0000000000001675. PMID: 33021569 PMC12755848

[101] Coudereau R Bodinier M Lukaszewicz A-C Py BF Argaud L Cour M . Persistent NLRP3 inflammasome activation is associated with delayed immunosuppression in septic patients. J Leukocyte Biol. (2024) 115:706–13. doi: 10.1093/jleuko/qiad161. PMID: 38146798

[102] Patil NK Bohannon JK Sherwood ER . Immunotherapy: A promising approach to reverse sepsis-induced immunosuppression. Pharmacol Res. (2016) 111:688–702. doi: 10.1016/j.phrs.2016.07.019. PMID: 27468649 PMC5026606

[103] Biswas SK Lopez-Collazo E . Endotoxin tolerance: New mechanisms, molecules and clinical significance. Trends Immunol. (2009) 30:475–87. doi: 10.1016/j.it.2009.07.009. PMID: 19781994

[104] O’Neill LAJ Pearce EJ . Immunometabolism governs dendritic cell and macrophage function. J Exp Med. (2016) 213:15–23. doi: 10.1084/jem.20151570. PMID: 26694970 PMC4710204

[105] Hsu CG Li W Sowden M Lage Chávez C Berk BC . Pnpt1 mediates NLRP3 inflammasome activation by MAVS and metabolic reprogramming in macrophages. Cell Mol Immunol. (2023) 20:131–42. doi: 10.1038/s41423-022-00962-2. PMID: 36596874 PMC9886977

[106] Luo R Li X Wang D . Reprogramming macrophage metabolism and its effect on NLRP3 inflammasome activation in sepsis. Front Mol Biosci. (2022) 9:917818. doi: 10.3389/fmolb.2022.917818. PMID: 35847986 PMC9276983

[107] Tsujimoto K Jo T Nagira D Konaka H Park JH Yoshimura S . The lysosomal Ragulator complex activates NLRP3 inflammasome *in vivo* via HDAC6. EMBO J. (2023) 42:e111389. doi: 10.15252/embj.2022111389. PMID: 36444797 PMC9811619

[108] Wang L Zhao M . Suppression of NOD-like receptor protein 3 inflammasome activation and macrophage M1 polarization by hederagenin contributes to attenuation of sepsis-induced acute lung injury in rats. Bioengineered. (2022) 13:7262–76. doi: 10.1080/21655979.2022.2047406. PMID: 35266443 PMC9208453

[109] Gritte RB Souza-Siqueira T Da Silva EB Dos Santos De Oliveira LC Borges RC De Oliveira Alves HH . Evidence for monocyte reprogramming in a long-term postsepsis study. Crit Care Explor. (2022) 4:e0734. doi: 10.1097/CCE.0000000000000734. PMID: 35928539 PMC9345639

[110] Guo H Ting JP-Y . Inflammasome assays *in vitro* and in mouse models. Curr Protoc Immunol. (2020) 131:e107. doi: 10.1002/cpim.107. PMID: 33017103 PMC7751762

[111] Ren W Rubini P Tang Y Engel T Illes P . Inherent P2X7 receptors regulate macrophage functions during inflammatory diseases. Int J Mol Sci. (2021) 23:232. doi: 10.3390/ijms23010232. PMID: 35008658 PMC8745241

[112] Wang L Zhao H Xu H Liu X Chen X Peng Q . Targeting the TXNIP‐NLRP3 interaction with PSSM1443 to suppress inflammation in sepsis‐induced myocardial dysfunction. J Cell Physiol. (2021) 236:4625–39. doi: 10.1002/jcp.30186. PMID: 33452697

[113] De Oliveira IM Chaves MM . The NLRP3 inflammasome in inflammatory diseases: Cellular dynamics and role in granuloma formation. Cell Immunol. (2025) 411–412:104961. doi: 10.1016/j.cellimm.2025.104961. PMID: 40339528

[114] Jiang N Li Z Luo Y Jiang L Zhang G Yang Q . Emodin ameliorates acute pancreatitis-induced lung injury by suppressing NLRP3 inflammasome-mediated neutrophil recruitment. Exp Ther Med. (2021) 22:857. doi: 10.3892/etm.2021.10289. PMID: 34178130 PMC8220649

[115] Kui L Kim AD Onyuru J Hoffman HM Feldstein AE . BRP39 regulates neutrophil recruitment in NLRP3 inflammasome-induced liver inflammation. Cell Mol Gastroenterol Hepatol. (2024) 17:481–97. doi: 10.1016/j.jcmgh.2023.12.002. PMID: 38092312 PMC10837621

[116] Hsu CG Lage Chávez C Zhang C Sowden M Yan C Berk BC . The lipid peroxidation product 4-hydroxynonenal inhibits NLRP3 inflammasome activation and macrophage pyroptosis. Cell Death Differ. (2022) 29:1790–803. doi: 10.1038/s41418-022-00966-5. PMID: 35264781 PMC9433404

[117] Hsu ML Jhuang KF Zouali M . Inflammasome functional activities in B lymphocytes. Immunol Res. (2024) 72:828–40. doi: 10.1007/s12026-024-09490-9. PMID: 38777958

[118] Lian X Guo Z Liu J Zeng W . Aerobic exercise affected lymphocyte apoptosis by modulating ROS release and NLRP3 inflammasome activation. Bull Exp Biol Med. (2025) 178:685–90. doi: 10.1007/s10517-025-06398-8. PMID: 40299126

[119] Huangfu L Wang J Li D Fei H Chen X Dong J . Fraxetin inhibits IKKβ, blocks NF-κB pathway and NLRP3 inflammasome activation, and alleviates spleen injury in sepsis. Chem Biol Interact. (2025) 408:111406. doi: 10.1016/j.cbi.2025.111406. PMID: 39921189

[120] Zhong Y Lu Y Yang X Tang Y Zhao K Yuan C . The roles of NLRP3 inflammasome in bacterial infection. Mol Immunol. (2020) 122:80–8. doi: 10.1016/j.molimm.2020.03.020. PMID: 32305691

[121] Babuta M Thevkar Nagesh P Datta AA Remotti V Zhuang Y Mehta J . Combined insults of a MASH diet and alcohol binges activate intercellular communication and neutrophil recruitment via the NLRP3-IL-1β axis in the liver. Cells. (2024) 13:960. doi: 10.3390/cells13110960. PMID: 38891092 PMC11171595

[122] Shao Y Li Y Jiang Y Li H Wang J Zhang D . Circulating exosomal miR‐155‐5p contributes to severe acute pancreatitis‐associated intestinal barrier injury by targeting SOCS1 to activate NLRP3 inflammasome‐mediated pyroptosis. FASEB J. (2023) 37:e23003. doi: 10.1096/fj.202300237R. PMID: 37219532

[123] Liang M Chen X Wang L Qin L Wang H Sun Z . Cancer-derived exosomal TRIM59 regulates macrophage NLRP3 inflammasome activation to promote lung cancer progression. J Exp Clin Cancer Res. (2020) 39:176. doi: 10.1186/s13046-020-01688-7. PMID: 32867817 PMC7457778

[124] Mezzasoma L Bellezza I Orvietani P Manni G Gargaro M Sagini K . Amniotic fluid stem cell‐derived extracellular vesicles are independent metabolic units capable of modulating inflammasome activation in THP‐1 cells. FASEB J. (2022) 36:e22218. doi: 10.1096/fj.202101657R. PMID: 35218567

[125] Peng Z Gong Z Wang Z Deng B Zhang X Lin J . Salvia miltiorrhiza -derived exosome-like nanoparticles improve diabetic cardiomyopathy by inhibiting NLRP3 inflammasome-mediated macrophage pyroptosis via targeting the NEDD4/SGK1 axis. Nanomedicine. (2025) 20:1417–28. doi: 10.1080/17435889.2025.2506351. PMID: 40391625 PMC12143680

[126] Shen J Wu J-M Hu G-M Li M-Z Cong W-W Feng Y-N . Membrane nanotubes facilitate the propagation of inflammatory injury in the heart upon overactivation of the β-adrenergic receptor. Cell Death Dis. (2020) 11:958. doi: 10.1038/s41419-020-03157-7. PMID: 33161415 PMC7648847

[127] Viola M Bebelman MP Maas RGC De Voogt WS Verweij FJ Seinen CS . Hypoxia and TNF‐alpha modulate extracellular vesicle release from human induced pluripotent stem cell‐derived cardiomyocytes. J Extracell Vesicles. (2024) 13:e70000. doi: 10.1002/jev2.70000. PMID: 39508403 PMC11541862

[128] Scalavino V Piccinno E Valentini AM Schena N Armentano R Giannelli G . miR-369-3p modulates intestinal inflammatory response via BRCC3/NLRP3 inflammasome axis. Cells. (2023) 12:2184. doi: 10.3390/cells12172184. PMID: 37681916 PMC10486421

[129] Chen K-T Yeh C-T Yadav VK Pikatan NW Fong I-H Lee W-H . Notopterol mitigates IL-1β-triggered pyroptosis by blocking NLRP3 inflammasome via the JAK2/NF-kB/hsa-miR-4282 route in osteoarthritis. Heliyon. (2024) 10:e28094. doi: 10.1016/j.heliyon.2024.e28094. PMID: 38532994 PMC10963379

[130] Al-Hawary SIS Jasim SA Romero-Parra RM Bustani GS Hjazi A Alghamdi M . NLRP3 inflammasome pathway in atherosclerosis: Focusing on the therapeutic potential of non-coding RNAs. Pathol Res Pract. (2023) 246:154490. doi: 10.1016/j.prp.2023.154490. PMID: 37141699

[131] Kodi T Sankhe R Gopinathan A Nandakumar K Kishore A . New insights on NLRP3 inflammasome: Mechanisms of activation, inhibition, and epigenetic regulation. J Neuroimmune Pharmacol. (2024) 19:7. doi: 10.1007/s11481-024-10101-5. PMID: 38421496 PMC10904444

[132] van Hout GPJ Bosch L Ellenbroek GHJM de Haan JJ van Solinge WW Cooper MA . The selective NLRP3-inflammasome inhibitor MCC950 reduces infarct size and preserves cardiac function in a pig model of myocardial infarction. Eur Heart J. (2017) 38:828–36. doi: 10.1093/eurheartj/ehw247. PMID: 27432019

[133] Jing W Lo Pilato J Kay C Feng S Enosi Tuipulotu D Mathur A . Clostridium septicum α-toxin activates the NLRP3 inflammasome by engaging GPI-anchored proteins. Sci Immunol. (2022) 7:eabm1803. doi: 10.1126/sciimmunol.abm1803. PMID: 35594341

[134] Zheng Y Zhang X Wang Z Zhang R Wei H Yan X . MCC950 as a promising candidate for blocking NLRP3 inflammasome activation: A review of preclinical research and future directions. Arch Pharm. (2024) 357:e2400459. doi: 10.1002/ardp.202400459. PMID: 39180246

[135] Nandi D Debnath M Forster J Pandey A Bharadwaj H Patel R . Nanoparticle-mediated co-delivery of inflammasome inhibitors provides protection against sepsis. Nanoscale. (2024) 16:4678–90. doi: 10.1039/D3NR05570A. PMID: 38317511

[136] Qin Y Li W Liu J Wang F Zhou W Xiao L . Andrographolide ameliorates sepsis-induced acute lung injury by promoting autophagy in alveolar macrophages via the RAGE/PI3K/AKT/mTOR pathway. Int Immunopharmacol. (2024) 139:112719. doi: 10.1016/j.intimp.2024.112719. PMID: 39032470

[137] Lian D Liu J Han R Jin J Zhu L Zhang Y . Kakonein restores diabetes‐induced endothelial junction dysfunction via promoting autophagy‐mediated NLRP 3 inflammasome degradation. J Cell Mol Med. (2021) 25:7169–80. doi: 10.1111/jcmm.16747. PMID: 34180143 PMC8335672

[138] Zhang X Hu Y Wang W Ji R Li Z Yu W . IRGM/Irgm1 increases autophagy to inhibit activation of NLRP3 inflammasome in inflammatory injury induced acute liver failure. Cell Death Discov. (2024) 10:272. doi: 10.1038/s41420-024-02052-w. PMID: 38849356 PMC11161524

[139] Deng W Yang Z Yue H Ou Y Hu W Sun P . Disulfiram suppresses NLRP3 inflammasome activation to treat peritoneal and gouty inflammation. Free Radical Biol Med. (2020) 152:8–17. doi: 10.1016/j.freeradbiomed.2020.03.007. PMID: 32151746

[140] Chen P Wang Y Tang H Zhou C Liu Z Gao S . New applications of clioquinol in the treatment of inflammation disease by directly targeting arginine 335 of NLRP3. J Pharm Anal. (2025) 15:101069. doi: 10.1016/j.jpha.2024.101069. PMID: 39902456 PMC11788862

[141] Xu H-W Li W-F Hong S-S Shao J-J Chen J-H Chattipakorn N . Tabersonine, a natural NLRP3 inhibitor, suppresses inflammasome activation in macrophages and attenuate NLRP3-driven diseases in mice. Acta Pharmacol Sin. (2023) 44:1252–61. doi: 10.1038/s41401-022-01040-z. PMID: 36627344 PMC10203108

[142] Saavedra-Torres JS Pinzón-Fernández MV Ocampo-Posada M Nati-Castillo HA Jiménez Hincapie LA Cadrazo-Gil EJ . Inflammasomes and signaling pathways: Key mechanisms in the pathophysiology of sepsis. Cells. (2025) 14:930. doi: 10.3390/cells14120930. PMID: 40558557 PMC12191029

[143] Ge Y Chen J Hu Y Chen X Huang M . IL‐38 alleviates inflammation in sepsis in mice by inhibiting macrophage apoptosis and activation of the NLRP3 inflammasome. Mediators Inflammation. (2021) 2021:6370911. doi: 10.1155/2021/6370911. PMID: 34955683 PMC8709774

[144] Alves GF Stoppa I Aimaretti E Monge C Mastrocola R Porchietto E . ICOS-Fc as innovative immunomodulatory approach to counteract inflammation and organ injury in sepsis. Front Immunol. (2022) 13:992614. doi: 10.3389/fimmu.2022.992614. PMID: 36119089 PMC9479331

[145] Ren J Lei G Dong A Cao S Han X Li H . Therapeutic potential of ADSC-derived exosomes in acute lung injury by regulating macrophage polarization through IRF7/NLRP3 signaling. Int Immunopharmacol. (2025) 156:114658. doi: 10.1016/j.intimp.2025.114658. PMID: 40252464

[146] Tang X Zeng T Deng W Zhao W Liu Y Huang Q . Gut microbe-derived betulinic acid alleviates sepsis-induced acute liver injury by inhibiting macrophage NLRP3 inflammasome in mice. mBio. (2025) 16:e0302024. doi: 10.1128/mbio.03020-24. PMID: 39887250 PMC11898617

[147] Huang W Wang X Xie F Zhang H Liu D . Serum NLRP3: A biomarker for identifying high-risk septic patients. Cytokine. (2022) 149:155725. doi: 10.1016/j.cyto.2021.155725. PMID: 34634653

[148] Goswami DG Walker WE . Detection of blood cell surface biomarkers in septic mice. In: Walker WE , editor.Sepsis, vol. 2321 . Springer US, New York, NY (2021). p. 191–205. Methods in Molecular Biology. doi: 10.1007/978-1-0716-1488-4_17, PMID: 34048018

[149] Gao Y Chen H Wu R Zhou Z . AI-driven multi-omics profiling of sepsis immunity in the digestive system. Front Immunol. (2025) 16:1590526. doi: 10.3389/fimmu.2025.1590526. PMID: 40463386 PMC12131868

[150] Cajander S Kox M Scicluna BP Weigand MA Almansa Mora R Flohé SB . Profiling the dysregulated immune response in sepsis: overcoming challenges to achieve the goal of precision medicine. Lancet Respir Med. (2024) 12:305–22. doi: 10.1016/S2213-2600(23)00330-2. PMID: 38142698

[151] Zhang H Liao J Jin L Lin Y . NLRP3 inflammasome involves in the pathophysiology of sepsis-induced myocardial dysfunction by multiple mechanisms. Biomedicine Pharmacotherapy = Biomedecine Pharmacotherapie. (2023) 167:115497. doi: 10.1016/j.biopha.2023.115497. PMID: 37741253

[152] Berk BC Lage Chávez C Hsu CG . PDE10A inhibition reduces NLRP3 activation and pyroptosis in sepsis and nerve injury. Int J Mol Sci. (2025) 26(10):4498. doi: 10.3390/ijms26104498. PMID: 40429643 PMC12111586

[153] Chen P-R Li C-Y Yazal T Chen I-C Liu P-L Chen Y-T . Protective effects of nordalbergin against LPS-induced endotoxemia through inhibiting MAPK/NF-κB signaling pathway, NLRP3 inflammasome activation, and ROS production. Inflammation Res. (2024) 73:1657–70. doi: 10.1007/s00011-024-01922-4. PMID: 39052062

[154] Ucar M Celebi O Celebi D Baser S Guler MC Tanyeli A . Determination of the effect of pyocyanin and Saccharomyces boulardii on gut microbiota and TLR4/MyD88/NF-κB and NLRP3 signaling pathways in sepsis induced by cecal ligation and puncture in rats. BMC Infect Dis. (2025) 25:931. doi: 10.1186/s12879-025-11308-4. PMID: 40691769 PMC12281731

[155] Kucuk A Aksungur N Halici Z Tavaci T Ozkaraca M Tebrizi B . Sunitinib treatment reduces proinflammatory cytokine levels and mortality rates by suppressing the NLRP3 inflammasome signaling pathway in sepsis. Inflammopharmacology. (2025) 33:5435–50. doi: 10.1007/s10787-025-01862-3. PMID: 40728674

[156] Li M Tan J Zhang R Gong X Xie J Liu C . Sunitinib alleviates hepatic ischemia reperfusion injury by inhibiting the JAK2/STAT pathway and promoting the M2 polarization of macrophages. Immunopharmacol Immunotoxicol. (2024) 46:672–84. doi: 10.1080/08923973.2024.2390455. PMID: 39155607

[157] Xu H Xu H Li W Liang Z Luo W Sheng S . Modulating the NLRP3 inflammasome: Acitretin as a potential treatment for sepsis-induced acute lung injury. Int Immunopharmacol. (2025) 153:114504. doi: 10.1016/j.intimp.2025.114504. PMID: 40187888

[158] Choe J-Y Kim S-K . Clinical significance of serum NLRP3 levels in patients with chronic gouty arthritis. Joint Bone Spine. (2018) 85:257–8. doi: 10.1016/j.jbspin.2017.02.009. PMID: 28257803

[159] Mohamed HI ELMeneza SA El-Bagoury IMS . The role of nod-like receptor family pyrin domain-containing 3 (NLRP3) inflammasome in diagnosis of late onset neonatal sepsis. J Neonatal-Perinatal Med. (2022) 15:787–93. doi: 10.3233/NPM-210909. PMID: 36031909

[160] Li Q Feng H Wang H Wang Y Mou W Xu G . Licochalcone B specifically inhibits the NLRP3 inflammasome by disrupting NEK7‐NLRP3 interaction. EMBO Rep. (2022) 23:e53499. doi: 10.15252/embr.202153499. PMID: 34882936 PMC8811655

[161] Xiao F Jia Y Zhang S Liu N Zhang X Wang T . SLC25A3 negatively regulates NLRP3 inflammasome activation by restricting the function of NLRP3. J Biol Chem. (2024) 300:107233. doi: 10.1016/j.jbc.2024.107233. PMID: 38552738 PMC11067542

[162] Xu Y Wang Y Lu C Liu Z Lin Z Zhang X . NLRP3 inflammasome: A new perspective on revealing hotspots and trends in uric acid metabolism disorders through bibliometric analysis. Endocrine Metab Immune Disord - Drug Targets. (2025) 25. doi: 10.2174/0118715303376897250702230058. PMID: 40698684 PMC13559964

[163] Qiang R Li Y Dai X Lv W . NLRP3 inflammasome in digestive diseases: From mechanism to therapy. Front Immunol. (2022) 13:978190. doi: 10.3389/fimmu.2022.978190. PMID: 36389791 PMC9644028

[164] Miao M Yang Y Dai H . Current research status and future prospects of NLRP3 inflammasome in cardiovascular diseases: a bibliometric and visualization analysis. Front Cardiovasc Med. (2024) 11:1407721. doi: 10.3389/fcvm.2024.1407721. PMID: 39022620 PMC11253129

[165] Ni J Guan C Liu H Huang X Yue J Xiang H . Ubc13 promotes K63-linked polyubiquitination of NLRP3 to activate inflammasome. J Immunol. (2021) 206:2376–85. doi: 10.4049/jimmunol.2001178. PMID: 33893171

[166] Huang L Luo R Li J Wang D Zhang Y Liu L . β-catenin promotes NLRP3 inflammasome activation via increasing the association between NLRP3 and ASC. Mol Immunol. (2020) 121:186–94. doi: 10.1016/j.molimm.2020.02.017. PMID: 32244067

[167] Huang S Wan P Huang S Liu S Xiang Q Yang G . The APC10 subunit of the anaphase‐promoting complex/cyclosome orchestrates NLRP3 inflammasome activation during the cell cycle. FEBS Lett. (2021) 595:2463–78. doi: 10.1002/1873-3468.14181. PMID: 34407203

[168] Zhang H Zhao C Hong G Xiong W Xia J Dong R . Fatty acid oxidation contributed to NLRP3 inflammasome activation caused by N-nitrosamines co-exposure. Food Chem Toxicol. (2025) 202:115549. doi: 10.1016/j.fct.2025.115549. PMID: 40374002

[169] Kang J-H Lee S-B Seok J Kim D-H Ma G Park J . Novel activity of ODZ10117, a STAT3 inhibitor, for regulation of NLRP3 inflammasome activation. Int J Mol Sci. (2023) 24:6079. doi: 10.3390/ijms24076079. PMID: 37047051 PMC10094431

